# Fine mapping of stem rust resistance derived from soft red winter wheat cultivar AGS2000 to an NLR gene cluster on chromosome 6D

**DOI:** 10.1007/s00122-024-04702-0

**Published:** 2024-08-19

**Authors:** L. Rivera-Burgos, C. VanGessel, M. Guedira, J. Smith, D. Marshall, Y. Jin, M. Rouse, G. Brown-Guedira

**Affiliations:** 1 Plant Science Research Unit, USDA-ARS, North Carolina State University, Raleigh, NC 27695 USA; 2 Department of Crop and Soil Sciences, Colorado State University, Fort Collins, CO 80523 USA; 3 Department of Crop and Soil Sciences, North Carolina State University, Raleigh, NC 27695 USA; 4 Department of Plant Pathology, North Carolina State University, Raleigh, NC 27695 USA; 5 Cereal Disease Laboratory, USDA-ARS, University of Minnesota, St. Paul, MN 55108 USA; 6 Sugarcane Production Research Unit, USDA-ARS, Canal Point, FL 33438 USA

## Abstract

The *Puccinia graminis* f. sp. *tritici* (*Pgt*) Ug99-emerging virulent races present a major challenge to global wheat production. To meet present and future needs, new sources of resistance must be found. Identification of markers that allow tracking of resistance genes is needed for deployment strategies to combat highly virulent pathogen races. Field evaluation of a DH population located a QTL for stem rust (Sr) resistance, *QSr.nc-6D* from the breeding line MD01W28-08-11 to the distal region of chromosome arm 6DS where Sr resistance genes *Sr42*, *SrCad*, and *SrTmp* have been identified. A locus for seedling resistance to *Pgt* race TTKSK was identified in a DH population and an RIL population derived from the cross AGS2000 × LA95135. The resistant cultivar AGS2000 is in the pedigree of MD01W28-08-11 and our results suggest that it is the source of Sr resistance in this breeding line. We exploited published markers and exome capture data to enrich marker density in a 10 Mb region flanking *QSr.nc-6D*. Our fine mapping in heterozygous inbred families identified three markers co-segregating with resistance and delimited *QSr.nc-6D* to a 1.3 Mb region. We further exploited information from other genome assemblies and identified collinear regions of 6DS harboring clusters of NLR genes. Evaluation of KASP assays corresponding to our co-segregating SNP suggests that they can be used to track this Sr resistance in breeding programs. However, our results also underscore the challenges posed in identifying genes underlying resistance in such complex regions in the absence of genome sequence from the resistant genotypes.

**Supplementary Information:**

The online version contains supplementary material available at 10.1007/s00122-024-04702-0.

## Introduction

Wheat stem rust is caused by the fungal pathogen *Puccinia graminis* Pers. f. sp. *tritici* Eriks. & E. Henn. (*Pgt*) and has been among the most historically significant threats to wheat production worldwide. Stem rust epidemics declined in the late twentieth century after the introduction of widely effective stem rust resistance genes (*Sr*) such as *Sr31* to breeding programs around the world (Pretorius et al. 2000). This resistance was overcome in 1998 with the emergence in Uganda of a new race of *Pgt*, referred to as Ug99 or TTKSK (Singh et al. 2011; Pretorius et al. 2000). In the last two decades, evolved races forming the Ug99-race group have developed as resistance genes in new cultivars deployed against Ug99 were overcome (Singh et al. 2015). The race group has spread to 13 countries throughout eastern Africa and the Middle East and is virulent to wheat grown on over 80% of acreage worldwide. Management practices for stem rust include removing the alternate host barberry (*Berberis vulgaris*), fungicide application, and incorporating effective genetic resistance into wheat cultivars (Roelfs 1989). There are limited resources for fungicide applications in developing countries where Ug99 is affecting wheat production, leaving resistant cultivar development as the most effective and practical tool against the pathogen.

There have been 65 stem rust resistance genes (*Sr*) or alleles numerically designated as well as nine temporarily named resistance genes with an uncertain relationship to numerically designated loci (Yu et al. 2014). About half of these loci are effective against Ug99; however, many are not used in cultivar development because they are located on alien chromosome translocations having deleterious effects on agronomic performance. Most named *Sr* genes provide race-specific resistance throughout the plant’s life cycle. In contrast, adult plant resistance (APR) provides relatively limited effectiveness only at the adult plant growth stage. Six quantitative trait loci (QTL) have been identified associated with APR: *Sr2*, *Sr55* (*Lr67*/*Yr46*/*Pm46*), *Sr56*, *Sr57* (*Lr34*/*Yr18*/*Pm38*), *Sr58* (*Lr46*/*Yr29*/*Pm39*) (Yu et al. 2014), and *Sr63* (Mago et al. 2022).

An approach for developing durably resistant wheat cultivars is to incorporate multiple sources of resistance into a single background. This pyramiding of resistance genes would decrease the likelihood of resistance being overcome by high disease pressure and prolong the durability of resistance (Singh et al. 2011). Stacking of *Sr* genes requires accurate molecular markers to track the presence of QTL/genes in breeding lines. The eastern growing region of the USA has maintained high levels of stem rust resistance in winter wheat cultivars due to multiple qualitative resistance genes present on alien translocations including *Sr31, Sr24*, *Sr36*, and *Sr1RS*^*Amigo*^ (Olson et al. 2010; Jin and Singh 2006). The emergence of Ug99 derivatives TTKST and TTTSK in Africa as well as races such as TKTTF and TTRTF that have incited large-scale epidemics puts winter wheat in this growing region at risk (Singh et al. 2015; Jin et al. 2009; Patpour et al. 2016). Other sources of resistance may be present in winter wheat breeding programs that have remained undetected due to masking by other *Sr* genes and avirulent North American *Pgt* races. Identifying sources of resistance already present in hexaploid bread wheat (*Triticum aestivum* L., AABBDD) that do not carry deleterious effects has been a priority.

The soft red winter wheat (SRWW) breeding line MD01W28-08-11 and the cultivar AGS2000 display resistance to stem rust. The breeding line MD01W28-08-11 showed partial adult plant resistance to Ug99 stem rust races TTKSK, TTKST, and TTKTT in Kenyan field tests. The cultivar AGS2000, which is a parent of MD01W28-08-11, showed seedling resistance to Ug99 stem rust race TTKSK in growth chamber conditions. The objectives of this study were: (1) to investigate the genetic basis of the field resistance in MD01W28-08-11; (2) to establish the relationship between the resistance in MD01W28-08-11 and seedling resistance in AGS2000; (3) to fine map the resistance; and (4) to develop KASP markers for marker-assisted selection applications.

## Material and methods

### Plant material

A population of 248 double haploids (DH) lines was developed from the cross of the cultivar Coker 9553 (89M-4035A/Pioneer 2580; PI 643092) with line MD01W28-08-11 (AGS2000/USG3209, developed at University of Maryland) and designated as the CM population. DNA marker analysis indicates that the Ug99-resistant parent MD01W28-08-11 has the t1RS·1BL translocation having the *Sr31* resistance gene while the susceptible parent Coker 9553 does not. The F_1_ was sent to Heartland Plant Innovations (HPI, Manhattan, KS) to produce the DH population by the maize (*Zea mays*) pollination method. The seed of the DH lines in the CM population was increased by the USDA-ARS in Raleigh N.C. and used for field and seedling screening.

A population of 256 F_5:6_ recombinant inbred lines (RILs) was developed from the cross between susceptible soft red winter wheat (SRWW) cultivar LA95135 (PI 655291) and resistant cultivar AGS2000 (PI 619256). LA95135 (CL850643/PIO2548//Coker9877/3/FL302/Coker-762) was released by Louisiana State University in 2006 and the cultivar AGS2000 (PIO2555/PF84301//Florida 302) was cooperatively developed and released by the University of Georgia and the University of Florida (Johnson et al. 2002). The population was designated as the LA population, and it was subjected to seedling screening in the greenhouse.

### Field stem rust assessment

MD01W28-08-11, Coker 9553, and the CM population were evaluated for stem rust reaction in the field at the Kenya Agricultural and Livestock Research Organization (KALRO) in Njoro, Kenya (− 0.33972, 35.94371, 2160 m elevation) in 2015 and 2016. Each year, seedlings of the lines were vernalized for 6 weeks at 4 °C, then transplanted to the field in hill plots, spaced approximately 30 cm apart. Each CM line was planted once and the parents were replicated twice. The susceptible control cultivar, Jagger (PI 593688, Sears et al. 1997), was included in every 50 plots in the nursery to monitor the level of infection. In both years, a mixture of stem rust-susceptible local spring wheat cultivars was planted in a continuous strip (spreader rows) directly adjacent to each hill plot to provide adequate stem rust inoculum. No hill plot was more than 10 cm away from a spreader row. Plants in the spreader rows were inoculated at booting and heading growth stages by injecting urediniospores directly into stems to promote infection and spore production. Three races were used for field stem rust phenotyping which represented the most prevalent Ug99-race group members in that region. The inoculum in both years included the original Ug99-race TTKSK (avirulence/virulence of *Sr36, Tmp, 24/Sr5, 21, 9e, 7b, 11, 6, 8a, 9 g, 9b, 30, 17, 9a, 9d, 10, 31, 38, McN*), TTKST (TTKSK with virulence to *Sr24*), and TTKTT (TTKSK with virulence to *Sr24* and *SrTmp*).

Field stem rust evaluations were conducted twice in 2015 and four times in 2016 at roughly the grain-filling stage. Only one rating, chosen for optimal discrimination between phenotypes, was used each year for QTL analyses. In both years, lines were evaluated for infection response (IR) and severity. Infection responses were classified as, in increasing severity, R, MR, MS, and S (R = resistant, S = susceptible, M = moderately). Severity was assessed as percent symptomatic plant area, with 0 indicating immunity and 100 indicating full susceptibility and complete coverage (CIMMYT 1986).

A composite score was developed for each rating to quantify IR and severity for QTL analysis. The composite score was calculated by first assigning infection response values of 0.1 (R to MSMR), 0.5 (MS), or 0.9 (MSS to S). This transformation was modified from the scale used by Bajgain et al. (2015). Severity scores were then multiplied by the weighted infection type to obtain a single numerical value (composite score) derived from the two-part rating. A second numerical score specifically representing the IR ratings was assigned as follows; R = 0, RMR = 1, MR = 2, MRR = 2, MRMS = 3, MSMR = 3, MS = 6, MSS = 7, SMS = 8, and S = 9. Both composite and infection response scales were used for linkage mapping of field stem rust resistance.

### Seedling inoculation and assessment

Seeds of the parents, Triumph 64 (*SrTmp*), Norin 40 (*Sr42*), AC Cadillac (*SrCad*) (Table 1), CM lines, LA lines, and other wheat cultivars and breeding lines were planted for evaluation of seedling infection type (IT) using four *Pgt* races inside a biocontainment safety level 3 facility at the University of Minnesota in conjunction with the USDA-ARS Cereal Disease Laboratory, St. Paul, MN in 2019 and 2020. The CM lines were evaluated for response to races TTKSK (Ug99; isolate 04KEN156/04), TRTTF (06YEM34-1), QCCJB (01SD80A), and QTHJC (75ND717C), and the LA recombinant lines were assessed only for response to race TTKSK. The experiments consisted of two replications of each race. For each replication, five seeds per line were planted in plastic pots filled with vermiculite (Sun Gro Horticulture, Agawam, USA). The pots were placed in a greenhouse maintained at 19–22 °C with a 16-h photoperiod provided by supplemental lighting. Approximately 10 days after planting when the primary leaves had fully emerged and the second leaves were beginning to grow, the seedlings were inoculated with the *P. graminis* f. sp. *tritici* isolates. The isolates were derived from single pustules and retrieved from storage at − 80 °C (Rouse et al. 2011; Olivera et al. 2012; Newcomb et al. 2016). Gelatin capsules each containing 14 mg of urediniospores were placed in plastic bags and submerged in a water bath set at 45 °C for 15 min. A total of 0.75 ml of light-weight mineral oil (Soltrol 70; ConocoPhillips Inc., Houston, USA) was added to each capsule to suspend spores. The spore suspension was used to inoculate 48 wheat lines (240 plants total) using a custom rust inoculator (St. Paul Machine Shop, University of Minnesota, St. Paul, USA) pressurized by an air pump (30 kPa). Oil was allowed to evaporate for 15 min following inoculation before placing plants in dew chambers maintained at 22 °C with ultrasonic humidifiers (V5100NS, Vicks, Marlborough, USA) that supplied a cool mist to the chamber for 2 min every 15 min over 16 h without light. Then, high-pressure sodium vapor lamps (400W, LR217718, Kavita Canada Inc., Niagara on the Lake, Canada) illuminated the dew chambers that each possessed a transparent plastic roof allowing light penetration to the plants. Two hours later, the humidifiers were turned off and the doors of the dew chambers were opened to allow the plants to dry. Seedlings were then placed in a greenhouse maintained at 19–22 °C with a 16-h photoperiod provided by supplemental lighting. The IT was assessed 13–14 days after inoculation according to the 0– 4 scale developed by Stakman et al. (1962) where IT ‘0–2’ indicate resistance and IT ‘3’ and ‘4’ indicate susceptibility. Relatively smaller and larger rust pustule sizes for each IT class were noted by ‘−’ and ‘+’ symbols. All IT was recorded on each leaf with the most frequent IT observed listed first. Lines with heterogeneous IT among plants were denoted by using a ‘/’ symbol to separate IT recordings for the various plants. Lines with an infection type of 33- or lower were considered resistant and seedlings yielding a high infection type of 3–4 were considered susceptible.

**Table 1:** Seedling virulence profiles of *SrTmp*, *Sr42*, and *SrCad* previously reported and *SrA2K* reported in this study

Locus	Source	Stem rust race^a^					References
		TTKSK	RKQSC	QCCJB	QTHJF	TRTTF	
*SrTmp*	Triumph 64	R	R	R	R	S	Kassa et al. (2016)
*Sr42*	Norin 40	R	R	S	S	S	Kassa et al. (2016)
*SrCad*	AC Cadillac	R	R	–	–	R	Kassa et al. (2016)
*SrA2K*	MD01W28-08-11	R	–	R	R^b^	S	Current study
*SrA2K*	AGS2000	R	–	–	R^b^	S	Current study

^a^Reaction ‘R’ = resistant reaction, ‘S’ = susceptible reaction, and ‘–’ = *Pgt* race not tested

^b^*SrA2K* reaction based on QTHJC race that is common in the USA and slightly different from QTHJF

The seedling IT scores were transformed to a numerical scale where 0.0, 1.0, 2.0, 3.0, and 4.0 corresponded to IT scores ‘0,’ ‘1,’ ‘2,’ ‘3,’ and ‘4,’ respectively. When a ‘−’ or ‘ + ’ was observed, we subtracted or added 0.5, respectively.

### Marker development and high-resolution map construction

The CM and LA populations were subjected to genotyping by sequencing (GBS) for single nucleotide polymorphism (SNP) discovery according to Poland et al. (2012). DNA was extracted from tissue collected from 10-day-old plants using the sbeadex plant maxi kit (LGC Genomics LLC, Teddington, UK). Ninety-six individual samples were barcoded, pooled into a single library, and sequenced on an Illumina HiSeq 2500. Sequencing reads were aligned to the International Wheat Genome Sequencing Consortium (IWGSC) RefSeq v1.0 assembly (https://wheat-urgi.versailles.inra.fr/Seq-Repository/Assemblies) using the Burrows-Wheeler Aligner v.0.7.12 (Li and Durbin 2010). The alignment information was processed by TASSEL-5GBSv2 pipeline version 5.2.35 (Glaubitz et al. 2014) for SNP calling. The data were filtered to retain SNP with ≤ 20% missing data, ≥ 5% minor allele frequency, and ≤ 10% of heterozygous calls per marker. A total of 3156 SNP for the CM population and 3400 SNP for the LA population were used in linkage map construction, respectively. The genetic maps were constructed using the MSTmap algorithm on the R/ASMap, and the R/qtl packages for R (Broman et al. 2003; Taylor and Butler 2017). Filtering removed low-quality markers, with an excess of missing values (≥ 15%), segregation distortion (Chi-square test; alpha = 0.01), and co-located markers (duplicated marker information) before map construction. Recombination frequencies were estimated in centiMorgans (cM) using the Kosambi mapping function. For each linkage group, a recombination plot (Heatmap) was drawn using R and standard functions.

### Data analysis and QTL mapping

The field IR and seedling IT data of each race were subjected to analysis of variance (ANOVA) using ‘lme4’ and ‘lmerTest’ packages for R (Bates et al. 2015; Kuznetsova et al. 2017). Adjusted means were estimated using the ‘emmeans’ package for R (Length et al. 2020) and subjected to the Shapiro–Wilk normality test and Chi-square test of goodness of fit.

Quantitative trait loci (QTL) analysis was performed on the CM population using a composite interval mapping (CIM) approach with a significance LOD (logarithm of the odds) threshold for an alpha = 0.05 determined using 5000 permutations. The effects of significant QTL were estimated using the Haley–Knott regression method (function ‘fitqtl’) in the R/qtl package (Broman et al. 2003).

### Fine mapping the *QSr.nc-6D* interval

After performing QTL analysis using the CM population, we targeted the detected QTL on chromosome arm 6DS, designated as *QSr.nc-6D*, by identifying new polymorphisms (SNP) for the parent lines of the LA mapping population in a 10 Mbp region flanking the *Sr* locus. We searched and selected SNP every 1 Mbp from exome capture data of the parents (https://wheat.triticeaetoolbox.org/) to design Kompetitive Allele Specific PCR (KASP, Semagn et al. 2014) assays that were used to genotype the CM and LA populations. The marker data were added to the genetic maps and QTL analysis was performed using the CM and LA populations as described above (Broman et al. 2003).

Additionally, the KASP marker sequences reported by Kassa et al. (2016) were blasted against the IWSGC reference assembly RefSeq v1.0 of Chinese Spring using BLAST+ (Camacho et al. 2009). Polymorphic markers that co-located with the *QSr.nc-6D* interval were added to the LA genetic map. We also identified a SNP from the 90 K array database located in our region and polymorphic between the parents of the LA population (https://wheat.pw.usda.gov/cgi-bin/GG3/browse.cgi; Wang et al. 2014). After being converted into a KASP assay, data were collected and the marker was added to the LA genetic map.

After the initial mapping, fourteen RIL from the LA population that were assigned heterozygous genotypes for flanking markers in the *QSr.nc-6D* interval were identified. Twenty-four F_6:7_ individuals in each heterozygous inbred line were genotyped with KASP markers to identify individual heterozygous plants that were then selfed to produce a segregating population of 340 plants (F_7:8_). Plants were genotyped with KASP markers and progeny of recombinant plants were evaluated for resistance to *Pgt* race TTKSK. Additional heterozygous plants were selfed to produce a large segregating population of 1,950 plants (F_8:9_). Recombinant plants were identified by genotyping and their progeny was evaluated for stem rust reaction as described above.

### Differentiation among *QSr.nc-6D, SrCad, Sr42* and *SrTmp* loci

The *QSr.nc-6D* resistance gene is located in a region similar to the previously identified *Sr42* (Norin 40), *SrCad* (AC Cadillac), and *SrTmp* (Triumph 64) genes providing race-specific resistance. The marker sequences reported by Kassa et al. (2016) and Hiebert et al. (2016) were blasted to Chinese Spring RefSeq v1.0 to obtain their genomic positions for comparison with our candidate region. These markers and those developed for our fine mapping were evaluated on DNA of AGS2000, MD01W28-08-11, Triumph 64, AC Cadillac, AC Karma, Norin 61, Coker9553, LA95135, Chinese Spring, and Jagger. The nullisomic–tetrasomic line TA3157_CS_N6D-T6B was used as a control for genome specificity of the marker assays. In addition, stem rust reactions of the lines with different *Pgt* races were compared.

### Micro-collinearity analysis of the *QSr.nc-6D* region among hexaploid wheat genomes

The genomic coordinates nearest flanking markers to the *QSr.nc-6D* region were used to search against genome sequences of Chinese Spring RefSeq v1.0 (http://www.wheat-urgi.versailles.inra.fr/; IWGSC 2018) and wheat cultivars Julius, CDC Landmark, CDC Stanley, Jagger, ArinaLrFor, SY Mattis, LongReach Lancer, Mace, Norin61 (Walkowiak et al. 2020), Fielder (Sato et al. 2021), Renan (Aury et al. 2022), and Kariega (Athiyannan et al. 2022) using the TGT tool (Chen et al. 2020).

## Results

### Phenotypic assessment

**Fig. 1:**
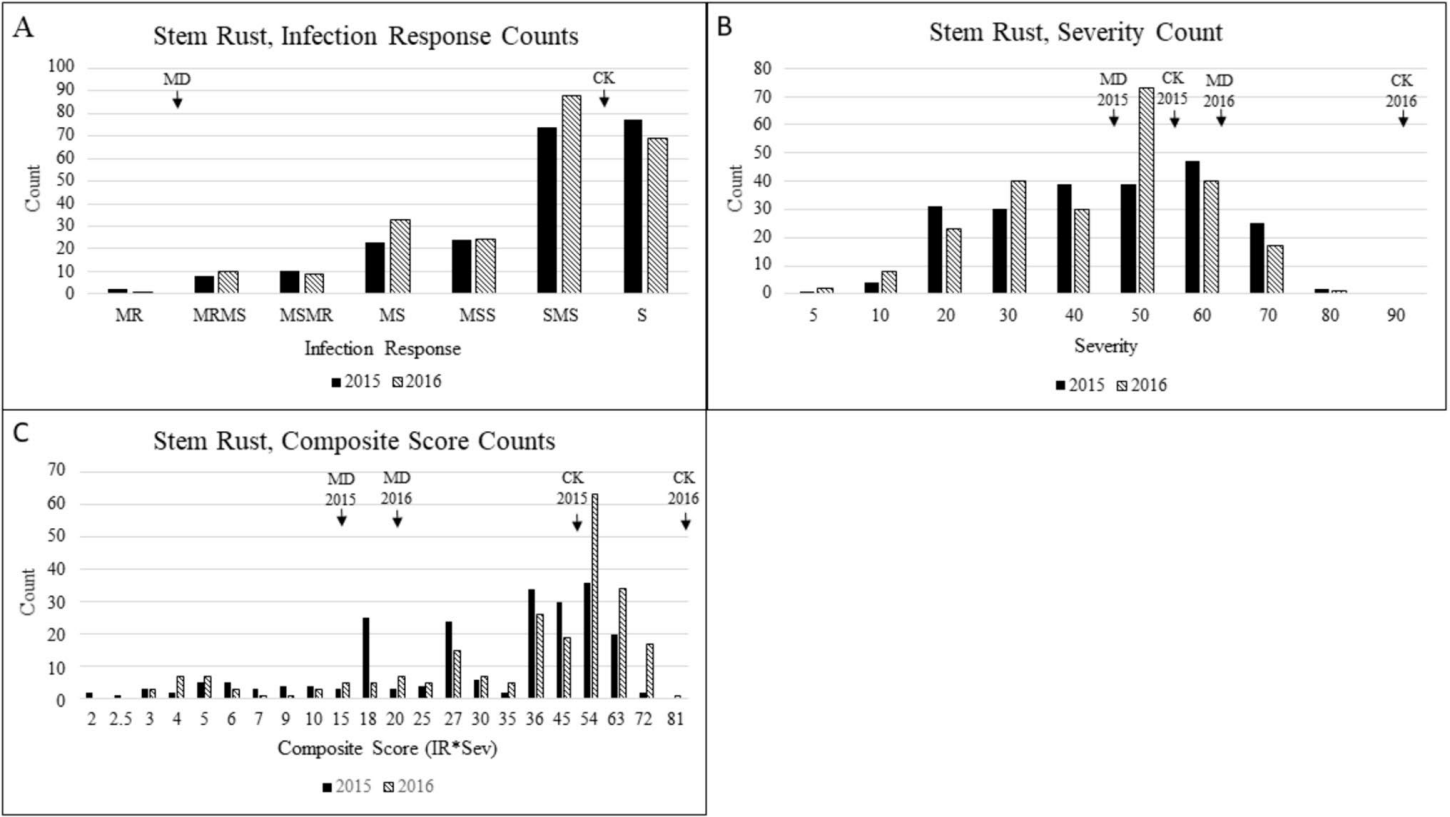
Distribution of stem rust infection response ratings, severity ratings, and composite scores on the double haploid Coker 9553/MD01W28-08-11 population in 2015 and 2016 in Njoro, Kenya. **A** Infection response (IR) ratings varying from MR to S (moderately resistant to susceptible), arrows indicate parental means across years. **B** Severity ratings (5 indicating 5% of affected leaf area, 90 indicating 90% of affected leaf area). **C** Composite score calculated by converted (0.1, 0.5, 0.9) infection type multiplied by severity; Arrows indicate mean parent phenotype; MD, MD01W28-08-11; CK, Coker 9553

Disease pressure was extremely high in Kenyan field evaluations in both 2015 and 2016 with all replications of the Jagger check having an IR of S, indicating minimal likelihood of escapes in the nursery. Coker 9553 was susceptible to Kenyan *P*. *graminis* f. sp. *tritici* races with IR of MSS and S while MD01W28-08-11 had moderately resistant IR of MR to MRMS (Fig. 1A). The severity scores were normally distributed in both years (Fig. 1B) and the composite ratings fell into discreet bins skewed toward resistance (Fig. 1C). The average composite score for the MD01W28-08-11 parent was 14.75 and 19.25 in 2015 and 2016, respectively, and 49.5 and 85.5 for the susceptible Coker 9553 parent (Fig. 1C).

Seedlings of the CM population were tested with *Pgt* race TTKSK, TRTTF, QCCJB, and QTHJC. Resistant DH lines showed IT 11- to 33-, whereas the susceptible lines showed IT 33+ to 4. The progeny segregated in a ratio not different from 1:1 (resistant/susceptible) indicating the presence of single genes, with different race specificity, in the CM population conferring resistance to *Pgt* races TTKSK (χ^2^_1:1_ = 1.77, 1 *d*.*f*., *P* = 0.184) and TRTTF (χ^2^_1:1_ = 3.88, 1 *d*.*f*., *P* = 0.050). For *Pgt* races QCCJB (χ^2^_1:1_ = 38.25, 1 *d*.*f*., *P* = 0.000) and QTHJC (χ^2^_1:1_ = 30.89, 1 *d*.*f*., *P* = 0.000), results indicated the presence of more than one gene segregating in the CM population (Table S1).

Seedlings of the LA population were tested with *Pgt* race TTKSK and the resistant RILs showed IT 11- to 33-, whereas the susceptible lines showed IT 33+ to 4. The progeny segregated in a ratio of 1:1 (resistant/susceptible) indicating the presence of a single gene conferring TTKSK resistance in the LA population (χ^2^_1:1_ = 2.83, 1 *d*.*f*., *P* = 0.0920; Table S1).

### Resistance gene mapping

The genetic map using 248 doubled haploid CM lines encompassed 2428 cM with 1249 loci assigned to 21 linkage groups corresponding to all 21 wheat chromosomes (Table S2). The LA population genetic map encompassed 4103 cM with 3406 SNP markers corresponding to 1881 loci. The map comprised 26 linkage groups assigned to all 21 wheat chromosomes, of which 1B, 4A, 5B, 6D, and 7A were more than one linkage group (Table S3).

**Fig. 2:**
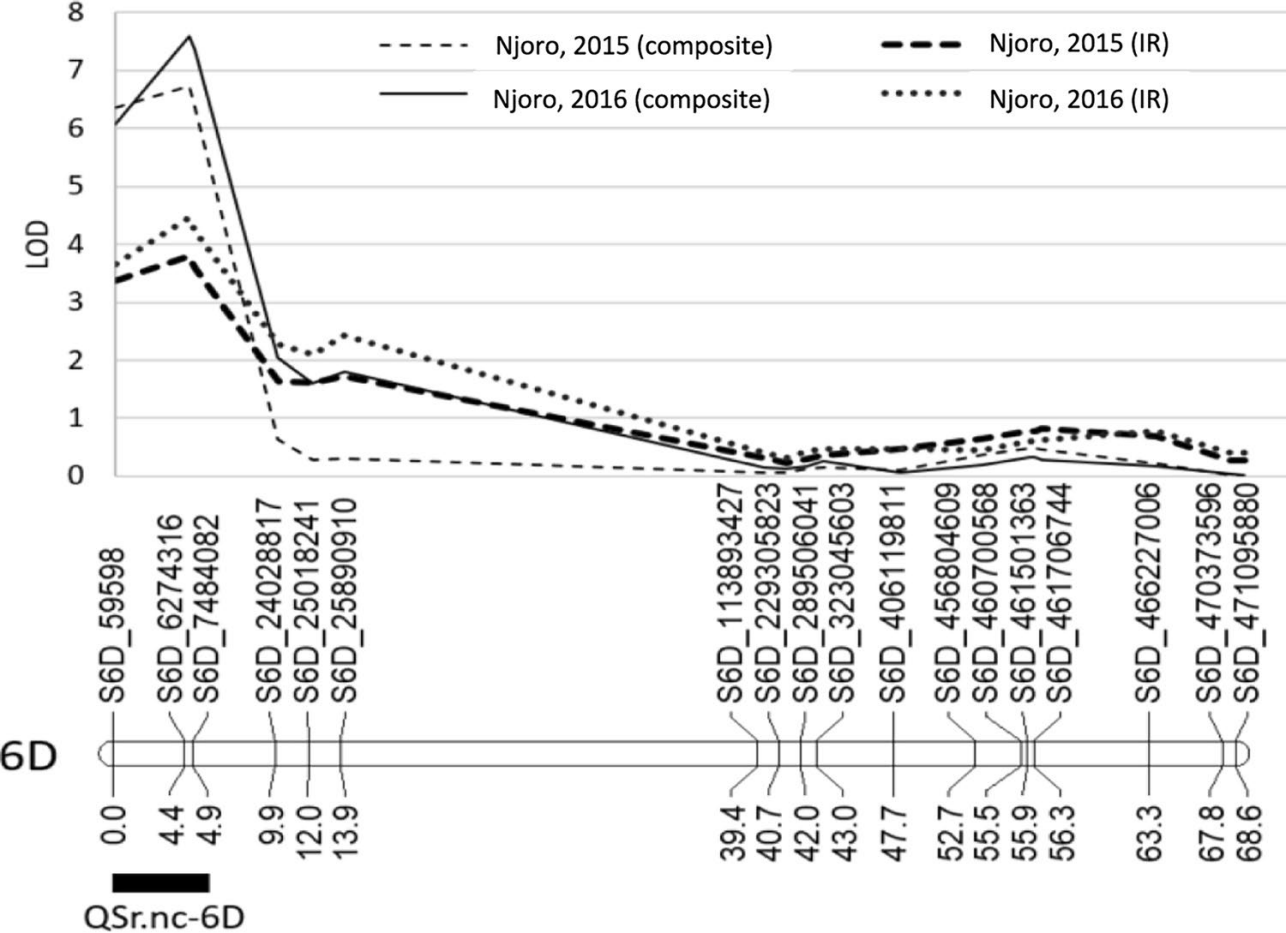
Quantitative trait loci (QTL) for resistance to stem rust identified on chromosome arm 6DS in Coker 9553/MD01W28-08-11 in Njoro 2015 and 2016; Composite and infection response (IR) rating scales used in linkage analysis are shown for each year

Analysis of the CM population identified QTL for field stem rust reaction in Njoro on the short arm of chromosome 6D and the short arm of chromosome 2B in both years and an additional QTL was detected on the long arm of chromosome 4B only in Njoro, 2016 (Table 2). The 6DS QTL (*QSr.nc-6D*) was stable and significant in both years of field screening for composite and IR rating methods (Table 2; Fig. 2). The LOD intervals for *QSr.nc-6D* for each evaluation were located at the most distal portion of the arm 6DS spanning from 0.0 to 4.9 cM with the peak LOD score at 4.5 cM. *QSr.nc-6D* explained ~ 13% of the composite scale variation in both years and 6.4 to 7.0% of IR variation in each year. The QTL peak on 2B was identified with the IR scale in both 2015 and 2016 and is designated *QSr.nc-2B* (Table 2). This QTL had a LOD interval that spanned approximately the same 15 cM region of 2BS in both years. *QSr.nc-2B* explained 8.5% and 7.4% of the variation in IR in 2015 and 2016, respectively. A QTL peak (*QSr.nc-4B*) on 4BL was identified for both composite and IR scales in 2016 but not in 2015 (Table 2). *QSr.nc-4B* explained 7.5% and 6.6% of the variation for composite and IR rating methods, respectively.

**Table 2:** Position and effect of quantitative trait loci (QTL) for field stem rust resistance based on interval mapping analysis of doubled haploid Coker 9553/MD01W28-08-11 population

Year/Location	Rating type^a^	QTL	LG^b^	QTL interval (cM)	Peak position (cM)	Physical position^c^ (bp)	LOD^d^	PVE^e^ (%)	Additive effect
2015 Njoro	Composite	*QSr.nc-6D*	6D	0.0–4.9	4.5	6,274,316	6.7	13.3	− 0.31
	IR	*QSr.nc-6D*	6D	0.0–4.6	4.5	6,274,316	3.8	6.4	− 0.12
		*QSr.nc-2B*	2B	42.3–57.4	50	63,135,770	5.0	8.5	− 0.14
2016 Njoro	Composite	*QSr.nc-6D*	6D	0.0–4.9	4.5	6,274,316	7.6	12.5	− 0.30
		*QSr.nc-4B*	4B	54.8–65.6	61.7	550,333,527	4.7	7.5	− 0.24
	IR	*QSr.nc-6D*	6D	0.0–4.9	4.5	6,274,316	4.5	7.0	− 0.12
		*QSr.nc-4B*	4B	54.8–65.6	65.6	635,685,764	3.3	6.6	− 0.11
		*QSr.nc-2B*	2B	42.3–57.8	50.8	66,011,112	4.2	7.4	− 0.12

^a^Scoring method used for linkage mapping analysis: IR = infection reaction (0–9), Composite = converted IR multiplied by severity

^b^Linkage group location of QTL

^c^QTL base pairs position in the Chinese Spring IWGSC RefSeq v1.0 genome assembly

^d^Logarithm of the odds at 0.05 level of probability obtained through a 5000-iteration permutation test

^e^Percentage variance explained by the QTL

**Fig. 3:**
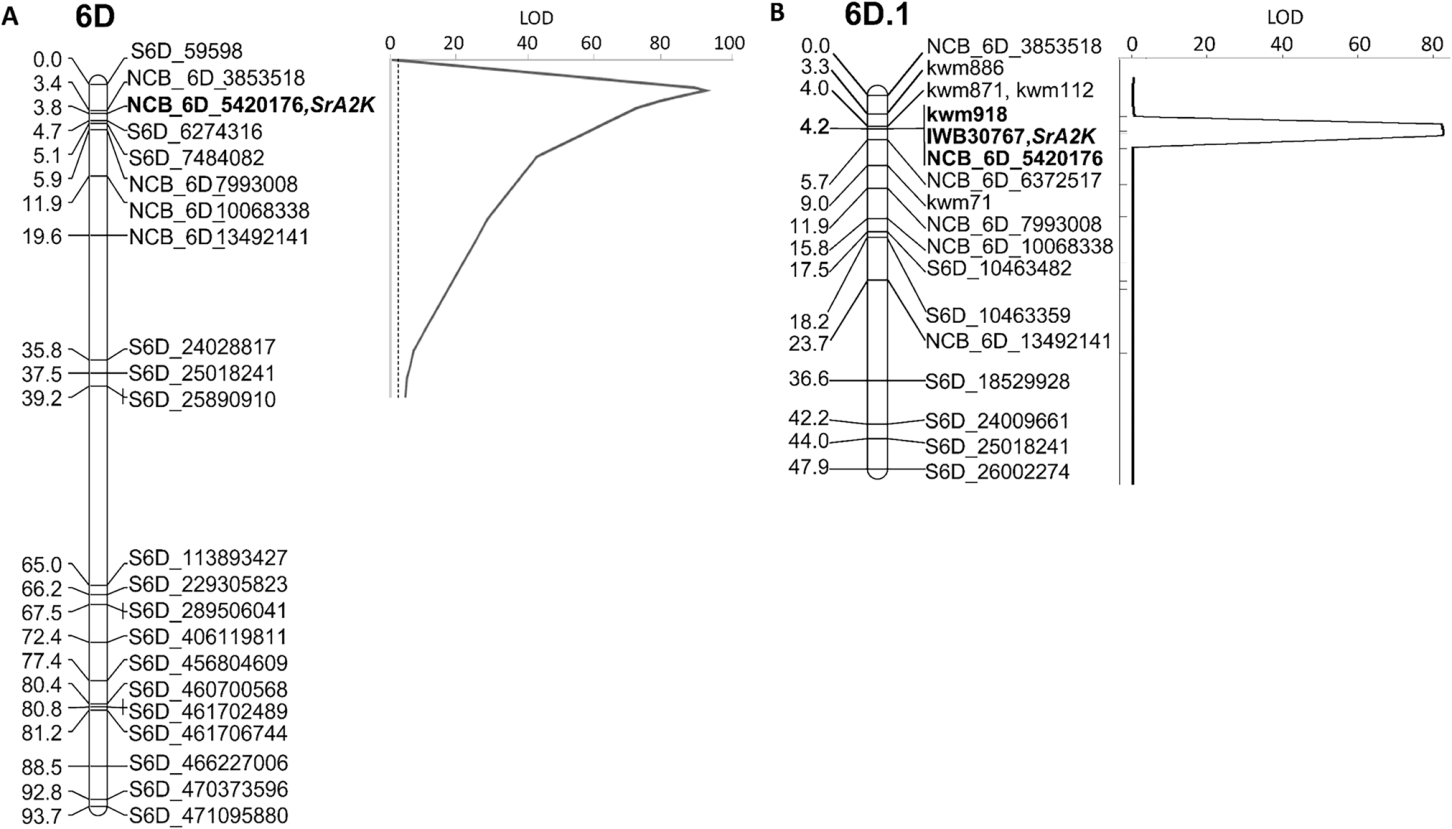
Quantitative trait loci (QTL) for seedling resistance to stem rust identified on linkage groups **A** 6D in the Coker 9553/MD01W28-08-11 DH population and **B** 6D.1 in the LA95135/AGS2000 RIL population using TTKSK race. In bold, KASP marker associated with the stem rust resistance in both populations. Prefixes NCB corresponds to KASP markers, IWB30767 is a 90 K SNP marker, S6D corresponds to GSB SNP marker, and kwm are markers reported by Kassa et al. (2016)

In the 2019 seedling evaluation, two QTL were identified in the CM population with resistance contributed by MD01W28-08-11 (Table 3). A QTL on the short arm of chromosome 1B contributed significant resistance to races QCCJB, QTHJC, and TRTTF. This QTL, postulated as *Sr31*, explained 21.9, 25.3, and 71.4% of the variation for these *Pgt* races in the CM population. A QTL on chromosome arm 6DS was identified for resistance to QCCJB, QTHJC, and TTKSK in the same region observed for field resistance in Kenya. Analysis of the LA population also identified QTL for stem rust resistance in linkage group 6D.1 that coincided with *QSr.nc-6D* in the CM population. For the TTKSK race, *QSr.nc-6D* explained ~ 81 and 77% of the IT variation in the CM and LA populations, respectively (Table 3; Fig. 3), suggesting the presence of a single gene in each population conferring seedling resistance to TTKSK hereafter referred to as *SrA2K.*

**Table 3:** Quantitative trait loci (QTL) associated with seedling stem rust resistance based on composite interval mapping of doubled haploid Coker 9553/MD01W28-08-11 and recombinant inbred line LA95135/AGS2000 populations when screened with *Pgt* races QCCJB, QTHJC, TRTTF, or TTKSK

Population^a^	*Pgt* race^b^	QTL	LG^c^	Peak position (cM)	Physical position^d^ (bp)	LOD^e^	PVE^f^ (%)	Additive effect
CM	QCCJB	*Sr31*	1B	7.2	146,796,395	20.3	21.9	− 0.23
		*QSr.nc-6D*	6D	4.6	6,274,316	17.8	30.2	− 0.26
	QTHJC	*Sr31*	1B	7.2	146,796,395	21.7	25.3	− 0.25
		*QSr.nc-6D*	6D	4.6	6,274,316	12.5	22.7	− 0.24
	TRTTF	*Sr31*	1B	7.2	146,796,395	62.0	71.4	− 0.42
	TTKSK	*QSr.nc-6D*	6D	4.6	6,274,316	82.5	81.3	− 0.46
LA	TTKSK	*QSr.nc-6D*	6D.1	3.0	5,420,176	84.0	73.9	− 0.32

^a^Coker9553/MD01W28-08-11 (CM) double haploid population; LA95135/AGS2000 (LA) RIL population

^b^*Puccinia graminis* f. sp. *tritici* races used for screening

^c^Linkage group location of QTL

^d^QTL base pairs position in the Chinese Spring IWGSC RefSeq v1.0 genome assembly

^e^Logarithm of the odds at 0.05 level of probability obtained through a 5000-iteration permutation test

^f^Percentage variance explained by the QTL

### High-resolution mapping of the *QSr.nc-6D* interval

**Fig. 4:**
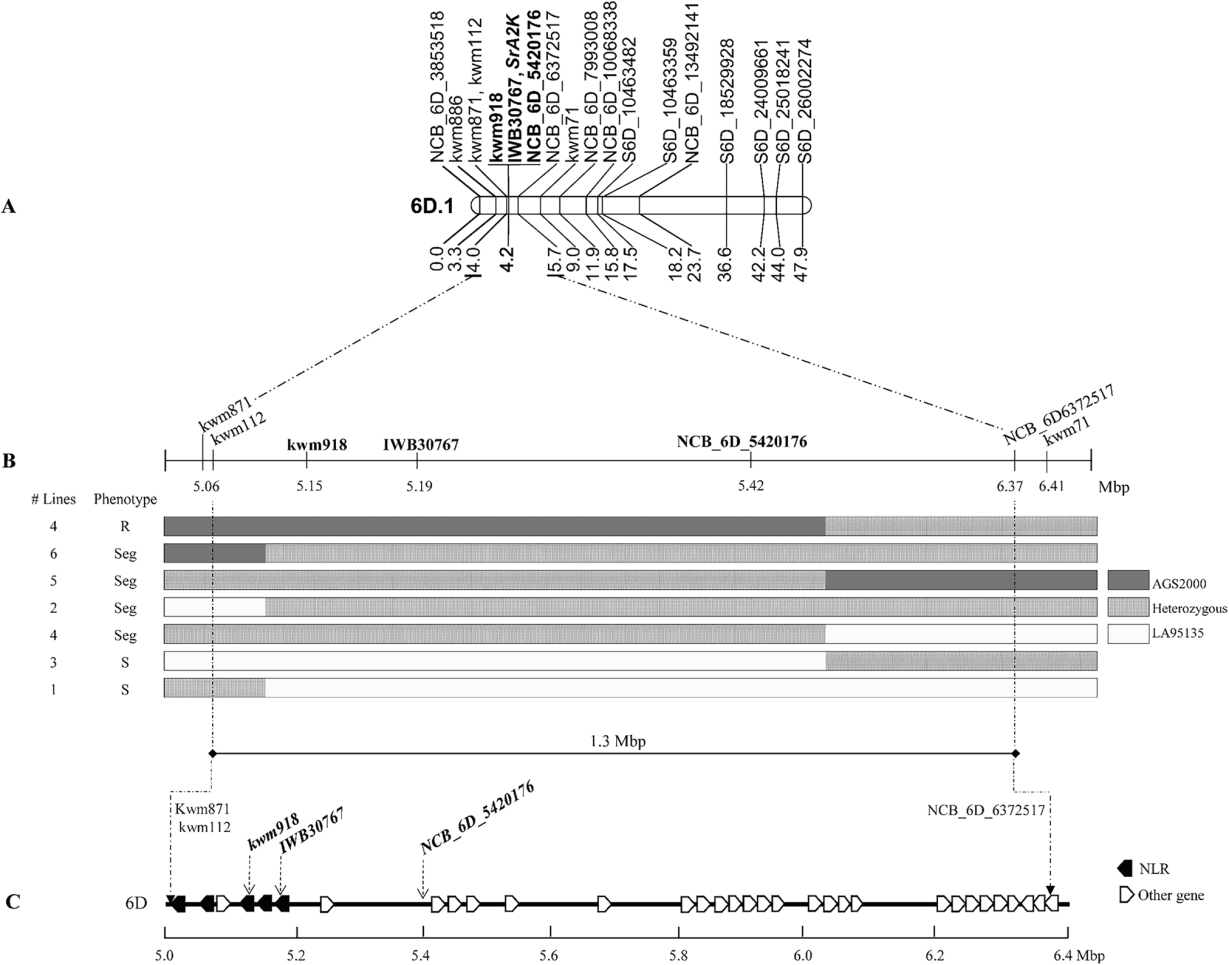
Fine mapping of stem rust resistance in soft red winter wheat cultivar AGS2000. **A** Genetic linkage map of *SrA2K* region. In bold, co-segregating KASP maskers. Numbers represent genetic distance in cM. **B** Phenotypes and genotypes of 25 heterozygous inbred lines displaying seven critical recombination events. Dark gray boxes indicate chromatin from AGS20000, light gray boxes indicate chromatin from LA95135, and dotted boxes indicate heterozygous segments. R, Seg, and S represent resistance, segregation, and susceptibility, respectively. **C** Annotated genes in the *SrA2K* region using the Chinese Spring IWGSC RefSeq v1.0 genome assembly. Dark pentagons indicate annotated NLRs. Co-segregating markers are indicated in bold

Assessment of data from exome capture of the parents of the LA population and polymorphism testing of previously reported markers linked to stem rust resistance loci located distally on the short arm of chromosome 6D identified polymorphic sites for KASP assay design in a 10 Mbp region flanking the *QSr.nc-6D* interval. Six assays targeting exome capture SNP and one SNP from the Illumina iSelect array (https://wheat.triticeaetoolbox.org; Wang et al. 2014), and five markers reported by Kassa et al. (2016) were evaluated on the LA mapping population (Table 4). The KASP markers kwm918, at Chinese Spring Ref v1.0 position 5,150,751 bp on chromosome 6D, IWB30767, at position 5,194,788 bp, and NCB_6D_5420176, at position 5,420,176 bp, co-segregated with IT data (Fig. 4a). When evaluated on the CM population, marker NCB_6D_5420176 co-segregated with resistance. Markers kwm918, IWB30767, and NCB_6D_5420176 also co-segregated with IT data when a total of 1984 progeny of selected LA RIL segregating in the QTL interval were genotyped with KASP markers. Evaluation of 25 recombinant families between flanking markers kwm871 and kwm71 for reaction to TTKSK, located the resistance gene in a physical interval of approximately 1.3 Mbp based on Chinese Spring Ref v1.0 genomic coordinates (Fig. 4b). The distal and proximal recombination breakpoints were between markers kwm112 and kwm918, and NCB_6D_5420176 and NCB_6D_6372517, respectively (Fig. 4b). We used the genomic coordinates of the KASP markers to search in the URGI database for annotated genes in the Chinese Spring wheat reference genome (https://wheat-urgi.versailles.inra.fr/Seq-Repository/Annotations). We identified 34 genes in the region spanned by the flanking markers, including an F-box gene and five NLR genes (Fig. 4c; Table S4).

**Table 4:** Kompetitive allele-specific PCR (KASP) markers used to fine map the *SrA2K* candidate region

Marker name^a^	SNP	RefSeq v1.0 6D position (bp)	Sequence^b^	
NCB_6D_3853518	C/T	3,853,518	AL1	ACTAGTAGCAGCACTCGATCCG
			AL2	AAACTAGTAGCAGCACTCGATCCA
			C1	CTTCGAGTTTGAAACGCCCGAGATT
kwm886	A/C	5,059,160	TAG1-	TCATTTGCCCAAATTTTGACCTGA
			TAG2-	TCATTTGCCCAAATTTTGACCTGC
			Rev-	AGTGTGAAGAATGGGATCGG
kwm871	A/G	5,064,480	TAG1-	GGCCATCATTATCTTCAGGATCTA
			TAG2-	GGCCATCATTATCTTCAGGATCTG
			Rev-	AACTTGGAGAGATCTATTTCAACAC
kwm112	C/T	5,064,580	TAG1-	ATGATATGGAGCATCACGACACAC
			TAG2-	CATGATATGGAGCATCACGACACAT
			Rev-	CTAATCTGTGAACCTTGTTGGTTGACTTT
kwm918	T/C	5,150,751	TAG1-	TCTTCCGTGAAGTGCTAATCTGT
			TAG2-	CTTCCGTGAAGTGCTAATCTGC
			Rev-	ACATGGCACAAGTTTGCGAGGACAA
IWB30767	G/T	5,194,788	AL1	CCGAGGATGTGGGCCGTG
			AL2	CCGAGGATGTGGGCCGTT
			C1	ATGCGACGTGTCCTTTCCTC
NCB_6D_5420176	C/T	5,420,176	AL1	CTCTCAACCTGTGAAACTCGTT
			AL2	CTCTCAACCTGTGAAACTCGTC
			C1	TCATCTACCTGGGTCTCTGGAA
NCB_6D_6372517	A/G	6,372,517	AL1	AGAGAAGTTTGGCCTATTGTGA
			AL2	AGAGAAGTTTGGCCTATTGTGG
			C1	GTCCTTTAAGAGAGGTAAGCCCATA
kwm71	C/T	6,342,756	TAG1-	GATGATGAACAAGTGGCCCCTC
			TAG2-	AAGATGATGAACAAGTGGCCCCTT
			Rev-	TTTACACAAGTTACACGCAAAACCGCATA
NCB_6D_7993008	G/A	7,993,008	AL1	GGTAATTCACATTAGTATTTTTTCCC
			AL2	AGCTGGTAATTCACATTAGTATTTTTTCCT
			C1	AGCATATGGAAGACATACTAT
NCB_6D_10068338	G/A	10,068,338	AL1	GCATTGGTGGAGCCGGTGTAG
			AL2	GCATTGGTGGAGCCGGTGTAA
			C1	TTTGGTCGGGCTCCGCTACCAT
NCB_6D_13492141	C/T	13,492,141	AL1	GGGAGTTCATCACGCACCTC
			AL2	GGGGAGTTCATCACGCACCTT
			C1	GGCATTGGAGAGCATGGCCCA

^a^NCB refers to North Carolina Brown-Guedira Laboratory, followed by the chromosome and physical position; IWB is a 90 K SNP-based KASP assay and kwm are markers reported by Kassa et al. (2016)

^b^KASP FRET cassette specific tail sequences omitted from primers TAG1-/TAG2- and AL1/AL2 for publication

### Comparative analysis of *SrA2K, SrCad, Sr42* and *SrTmp* loci

The *SrCad, Sr42,* and *SrTmp* loci have similar positions in chromosome 6D (Hiebert et al. 2016; Kassa et al. 2016) and are either alleles of the same gene or different genes tightly linked in repulsion. *SrCad, Sr42,* and *SrTmp* coincide with the location of *SrA2K* in this study and each of these loci confer resistance to *Pgt* Ug99-race TTKSK. Previous analysis with multiple *Pgt* races demonstrated differences in virulence profiles among *SrCad*, *Sr42*, and *SrTmp* (Table 1). Similar reactions to races TTKSK, QCCJB, QTHJC, and TRTTF were observed in our evaluation of MD01W28-08-11 and those reported for the cultivar Triumph 64 (*SrTmp*) by Kassa et al. (2016). Both lines showed resistance to *Pgt* races TTKSK, QCCJB, QTHJC, and susceptibility to TRTTF. Although Norin 40 (*Sr42*) showed resistance to TTKSK, it was susceptible to races QCCJB and QTHJC, distinguishing it from *SrTmp* and *SrA2K*. Likewise, resistance in cultivar AC Cadillac (*SrCad*) is distinguished from the other genes based on its resistant reaction to race TRTTF. Different marker haplotypes were observed when KASP assays developed in this study and those reported in Kassa et al. (2016) were compared for MD01W28-08-11, AGS2000, and lines having *SrTmp*, *Sr42,* and *SrCad* in addition to four susceptible cultivars (Table 5). The SNP markers kwm918, IWB30767, and NCB_6D_5420176 that co-segregated with resistance in our mapping populations distinguished the haplotype in AGS2000, MD01W28-08-11, and Triumph 64 from all resistant and susceptible lines evaluated. This unique marker haplotype in AGS2000, MD01W28-08-11 (*SrA2K*), and Triumph 64 (*SrTmp*) corresponded to a 0.3 Mbp genomic region in the Chinese Spring reference genome (RefSeq v1.0). Except for kwm112, the nullisomic–tetrasomic line, TA3157_CS_N6D-T6B, did not yield a PCR product, supporting the genome specificity of the designed KASP markers.

**Table 5:**
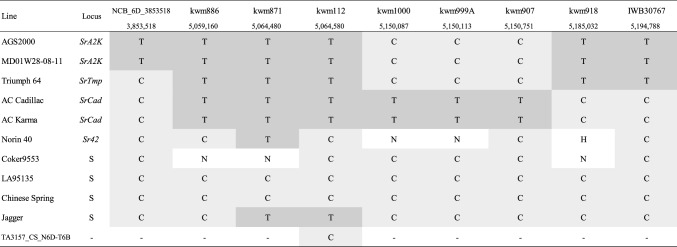
Haplotype of wheat lines with SNP markers linked to *SrA2K* and *SrCad/Sr42*

S indicates susceptible controls; NCB corresponds to exome capture KASP markers; kwm are markers reported by Kassa et al. (2016); kwm1000, kwm999A, and kwm907 are SNP markers linked to *SrCad* (Kassa et al. 2016); IWB is a 90 K SNP-based KASP assay; marker genomic positions (bp) are based on Chinese Spring IWGSC RefSeq v1.0 genome assembly; T indicates resistant allele; C indicates susceptible allele; N indicates no amplification; H indicates heterozygous

We tested the co-segregating KASP markers in a subset of 68 entries from the 2022 Eastern Soft Red Winter Wheat cooperative nurseries to assess marker predictability for marker-assisted selection purposes. Lines were evaluated using TTKSK and TTKTT *Pgt* races to postulate those having *SrTmp/SrA2K* and/or the *Sr24* gene. A low response to TTKSK and a higher response to TTKTT would indicate the presence of *SrTmp*/*SrA2K*. Of the 68 lines evaluated, 17 were classified as resistant, including the check cultivar AGS2000 (Table S5). Analysis with predictive marker assays indicated that five lines possessed *Sr24*, three had *Sr36*, and one line had the t1RS·1AL chromosome possessing *Sr1RS*^*Amigo*^. Of eight lines that displayed a resistance phenotype to TTKSK and did not possess *Sr24*, *Sr36*, or *Sr1RS*^*Amigo*^, six had the *SrA2K* resistance haplotype using markers kwm918, IWB30767_12, and NCB_6D_5420176 (Table S5). Breeding lines GA161137LDH-23-20LE3 and LA15093SB-30-2 were predicted to have both the *SrA2K* and *Sr24* resistance loci. These results suggest that our co-segregating KASP markers can be used to track stem rust resistance *SrA2K* in breeding programs.

### Pangenome analysis of the *SrA2K* locus

**Fig. 5:**
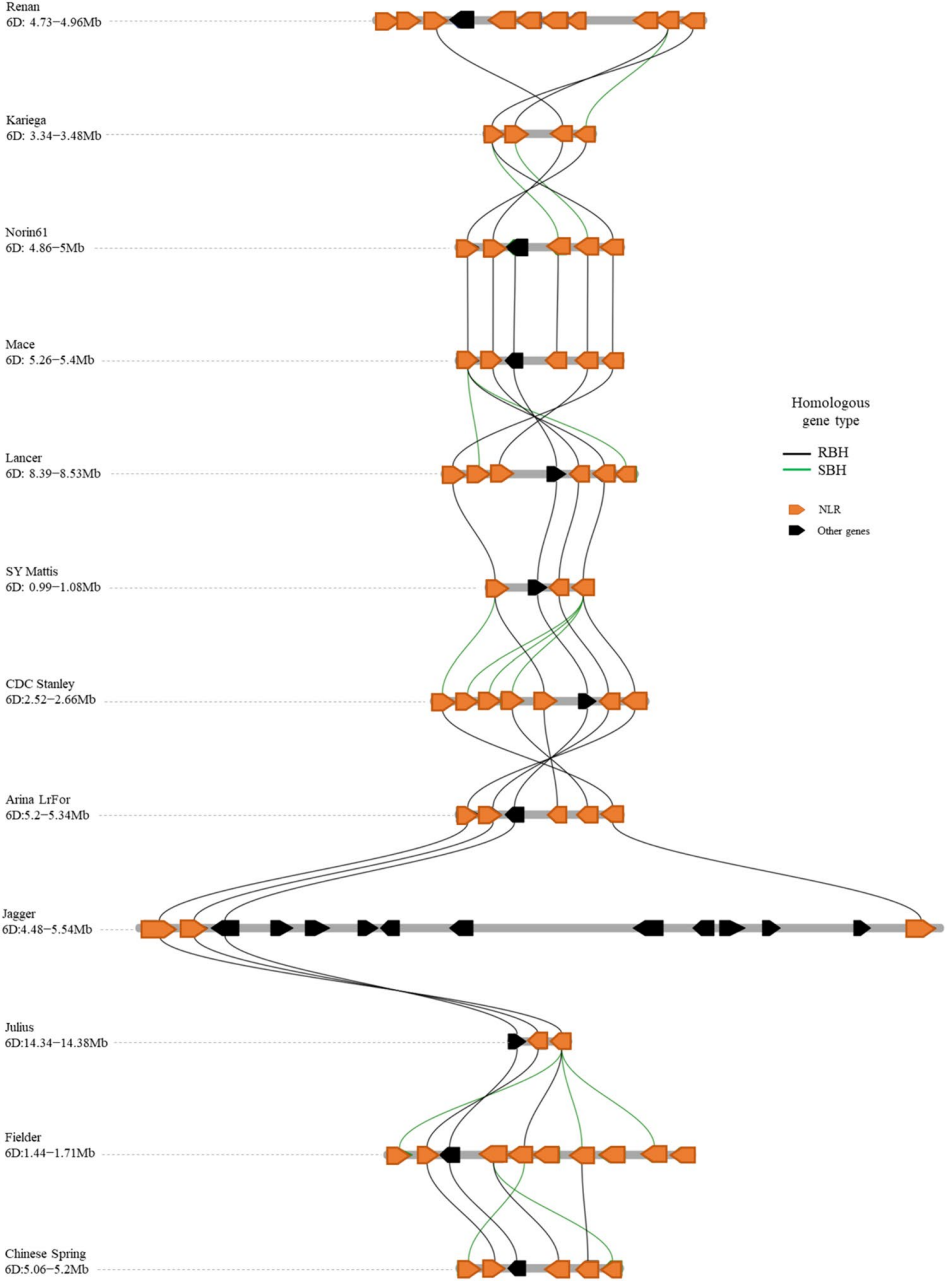
Micro-collinearity analysis of the identified NLR cluster in the *SrA2K* region between Chinese Spring and hexaploid wheat genomes (Chen et al. 2020). Orthology detection methods: RBH (Reciprocal Best Hit) and SBH (Single-side Best Hit)

With the genomic coordinates of flanking markers kwm871, kwm112, and NCB_6D6372517, and co-segregating makers kwm918, IWB30767, and NCB_6D_5420176, we delineated the *SrA2K* locus in the Chinese Spring reference sequence (RefSeq v1.0). A cluster of five NLR genes and one F-box protein was observed in chromosome arm 6DS in the 0.14 Mbp region between 5,058,868 and 5,198,817 bp (Table S4). BLAST was used to investigate the collinear relationship of this interval in Chinese Spring with other hexaploid wheat genome assemblies (Fig. 5). In the other published genomes, we identified between two to ten NLR genes and one gene with an unknown function. Similar to Chinese Spring, the collinear interval in Arina LrFor, Mace, and Norin61 displayed five NLR genes in a genomic interval of 0.14 Mbp. The collinear interval in LongReach Lancer, CDC Stanley, Fielder, and Renan had six, seven, nine, and ten predicted NLR genes, respectively, in interval lengths between 0.14 and 0.27 Mbp. The winter cultivars SY Mattis and Julius displayed three and two NLR genes in smaller interval lengths of 0.09 and 0.04 Mbp, respectively. The cultivar Kariega displayed four genes, all annotated as NLR genes. A more divergent interval of 1.06 Mbp was present in the winter wheat cultivar Jagger having three NLR genes and eleven genes with other or unknown functions (Table S6). These results suggest that stem rust resistance on chromosome 6D resides in a region having structural variation across the diverse wheat genomes examined.

## Discussion

Stem rust has been a sporadic disease in US winter wheat growing regions since 1956. Phenotyping for *Pgt* race TTKSK and derivatives is primarily limited to evaluations in collaborative screening done in Njoro, Kenya, and secure biocontainment facilities at the Cereal Disease Laboratory in St. Paul, MN. Olson et al. (2010) used molecular markers to demonstrate that high levels of stem rust resistance in eastern winter wheat were primarily due to *Sr31*, *Sr1RS*^*Amigo*^, *Sr24* and *Sr36*. The virulence of the Ug99-race group to *Sr31*, *Sr24*, and *Sr36* was considered particularly significant due to their widespread usage (Singh et al. 2015). Identifying new sources of resistance and stacking these with other effective resistance genes is necessary to develop new cultivars that can withstand heavy disease and selection pressure (Hiebert et al. 2011). This strategy requires the identification of robust DNA markers for the targeted resistance genes.

The MD01W28-08-11 line was notable for its moderate Ug99 stem rust resistance in Kenya screenings and our analyses suggest it possesses multiple QTL for resistance. However, *QSr.nc-4B* was detected only in 2016 and *QSr.nc-2B* was significant using the infection response rating in both years but not using the composite scale incorporating severity. We investigated *QSr.nc-2B* to determine whether it was a minor source of resistance or a physiological response. In order to screen winter wheat in Kenya close to the equator, lines are artificially vernalized prior to transplanting. This can lead to extreme phenotypes for maturity exacerbated by a climate and photoperiod for which varieties may not be adapted. Heading date data collected in Raleigh, N.C. indicated that the CM population was segregating for maturity and linkage mapping found that *QSr.nc-2B* closely corresponded to a heading date QTL on 2B (data not shown). This suggests that *QSr.nc-2B* may identify the photoperiod gene *Ppd-B1* segregating in the population and the IR score alone cannot distinguish maturity effects from *Pgt* resistance in the Kenya environment. The *QSr.nc-6D* was the most significant QTL in the CM population, explaining approximately 13% of the phenotypic variation in the composite score each year. The relatively small proportion of the phenotypic variation explained may be due to the confounding effects of maladaptation in the Kenya environment, field race composition, and heavy disease pressure. Operational constraints of screening germplasm in Ug99 stem rust inflicted regions allowed only two years of unreplicated phenotyping in Kenya; however, the field QTL on 6D co-located with seedling QTL and supports the relevance of this locus in high disease pressure agricultural settings. Seedling testing in 2009 and 2010 of MD01W28-08-11 showed IT of 2+/3, 2+, and 2+ when evaluated with TTKSK, TTKST, and TTTSK, respectively, indicating moderate levels of seedling resistance. Similar IT was observed for AGS2000, the likely parental source of resistance in MD01W28-08-11, and our analyses demonstrated that the resistance to TTKSK in AGS2000 was also mapped to the short arm of chromosome 6D. Thus, we focused on characterizing and fine mapping this stem rust resistance, referred to as *SrA2K*, and the development of predictive markers for MAS applications.

### Similarity of *SrA2K* and *SrTmp*

We delimited stem rust resistance in AGS2000 to an interval of 1.7 cM in the distal region of the short arm of chromosome 6D corresponding to ~ 1.3 Mb region in the Chinese Spring reference assembly (RefSeq v1.0). This region of 6DS has at least five previously reported stem rust loci: *Sr5*, *Sr42*, *SrTmp*, *SrCad*, and *SrTA10187* (Olson et al. 2013; Gao et al. 2015; Wiersma et al. 2016; Hiebert et al. 2016; Kassa et al. 2016). *Sr5* and *SrTA10187* are unlikely candidates for the resistance found in MD01W28-08-1 and AGS2000. *Sr5* does not provide resistance to Ug99. *SrTA10187* was recently introgressed into hexaploid wheat from the diploid progenitor *Aegilops tauschii* Coss. that does not appear in the pedigrees of our resistant parents.

Hiebert et al. (2016) compared the resistance in three mapping populations derived from cultivars Norin 40 (*Sr42;* Ghazvini et al. 2012), Triumph 64 (*SrTmp;* Mcvey and Hamilton 1985), and AC Cadillac (*SrCad;* Hiebert et al. 2011). Each resistance source showed similar resistant phenotypes of 12- to 2- in TTKSK seedling screening but had different resistance profiles to TRTTF, and North American races RKQSC, QCCJB, and QTHJF. (Hiebert et al. 2011; Ghazvini et al. 2012; Kassa et al. 2016). Resistance co-segregated with SNP markers on 6DS developed by Hiebert et al. (2016) and the physical positions of *Sr42*, *SrCad* and *SrTmp* could not be distinguished. However, the varying specificities to *Pgt* races of these QTL indicate separate genes or allelic variants are at play (Hiebert et al. 2016; Jin et al. 2007).

We assessed AGS2000 and MD01W28-08-11 with *Pgt* isolates synonymous with or closely related to the four isolates previously used to discriminate *Sr42*, *SrTmp,* and *SrCad* (Hiebert et al. 2016; Table 1). All lines showed similar IT scores after inoculation with TTKSK. The *SrA2K* locus was distinguished from *Sr42* by resistance to the QCCJB and QTHJC and from *SrCad* by having susceptibility to TRTTF. However, these seedling reaction types did not distinguish *SrA2K* and *SrTmp.* Similarly, the evaluation of these cultivars with co-segregating markers kwm918, IWB30767, and NCB_6D_5420176 differentiated *SrA2K* from *SrCad* and *Sr42* but not from *SrTmp*. Taken together, our data support the assertion that *SrA2K* differs from *SrCad*, and *Sr42*; however, *SrA2K* and *SrTmp* could be the same gene that confers resistance to stem rust race TTKSK. Further examination of this region of the 6D chromosome through targeted sequencing of candidate genes should shed light on the relationship of *SrA2K* with *SrTmp*, as well as other resistance genes in the region.

### Pangenome analysis identifies an NLR cluster on 6DS

Our fine mapping findings suggest that the genomic region of the *SrA2K* locus corresponds to 1.3 Mb where an F-box gene and a cluster of five NLR resistance genes have been annotated according to the Chinese Spring reference sequence (RefSeq v1.0). The pangenome analysis also identified clusters having two to ten 10 NLR genes in the region collinear to the *SrA2K* locus in the other hexaploid wheat genome assemblies (Fig. 5). A clustered genomic arrangement as a result of tandem duplication, unequal crossing over, and intra-cluster chromosomal rearrangement events is a common characteristic of evolving and well-diversified genes such as NLRs (Barragan and Weigel 2021). In an NLR wheat pangenome study, Andersen et al. (2020) found that more than 50% of the identified NLRs formed gene clusters. These clusters can be formed by a few or several NLRs. For instance, the powdery mildew resistance gene *MlWE74* was found in a cluster of three to five NLRs among hexaploid wheat genome assemblies (Zhu et al. 2022). In contrast, Li et al. (2022) reported that *Pm69* is located in a genomic region where more than 40 NLRs reside in clusters.

Understanding the sources of resistance present in the US wheat germplasm is critical for a better assessment of Ug99 resistance in North America. It will also help determine which cultivars are good candidates for stacking resistance genes in the background of elite germplasm. We fine-mapped an all-stage resistance gene in the chromosome arm 6DS, different from *Sr42* and *SrCad*. The 6DS candidate regions harbor a cluster of NLR genes in Chinese Spring and other hexaploid wheat genomes making it challenging to identify the gene underlying resistance in such a complex genomic region. However, our KASP markers track the stem rust resistance with high predictability making them an effective tool for MAS application with the goal of engineering and releasing durable high yielding-*Sr*-resistant cultivars.

## Supplementary Information

Below is the link to the electronic supplementary material.

Supplementary file1 (DOCX 88 KB)

## Data Availability

The data underlying this article are available in the article and its online supplementary material.
