# Supplementary material for: Fine mapping of stem rust resistance derived from soft red winter wheat cultivar AGS2000 to an NLR gene cluster on chromosome 6D
Source: Theor Appl Genet. 2024 Aug 19;137(9):206. doi: 10.1007/s00122-024-04702-0 (PMC11333525; doi:10.1007/s00122-024-04702-0)
Supplement: Supplementary file 1 — Supplementary file1 (DOCX 88 KB) [file 122_2024_4702_MOESM1_ESM.docx]

Table S 1. Single locus chi-square goodness-of-fit-test (P < 0.05) for resistance to stem rust in the CM and LA populations.

| Progeny | Resistant | Susceptible | Expected | ꭓ^2^value | df | *P* value |
| --- | --- | --- | --- | --- | --- | --- |
| CM population |  |  |  |  |  |  |
| TTKSK |  |  |  |  |  |  |
| MD01W28-08-11 | 5 | 0 |  |  |  |  |
| Coker9553 | 0 | 5 |  |  |  |  |
| DH Lines | 126 | 148 | 1:1 | 1.77 | 1 | 0.184 |
|  |  |  |  |  |  |  |
| TRTTF |  |  |  |  |  |  |
| MD01W28-08-11 | 5 | 0 |  |  |  |  |
| Coker9553 | 0 | 5 |  |  |  |  |
| DH Lines | 124 | 157 | 1:1 | 3.88 | 1 | 0.050 |
|  |  |  |  |  |  |  |
| QCCJB |  |  |  |  |  |  |
| MD01W28-08-11 | 5 | 0 |  |  |  |  |
| Coker9553 | 0 | 5 |  |  |  |  |
| DH Lines | 187 | 85 | 1:1 | 38.25 | 1 | 0.000 |
|  |  |  |  |  |  |  |
| QTHJC |  |  |  |  |  |  |
| MD01W28-08-11 | 5 | 0 |  |  |  |  |
| Coker9553 | 0 | 5 |  |  |  |  |
| DH Lines | 183 | 91 | 1:1 | 30.89 | 1 | 0.000 |
|  |  |  |  |  |  |  |
| LA population |  |  |  |  |  |  |
| TTKSK |  |  |  |  |  |  |
| AGS2000 | 5 | 0 |  |  |  |  |
| LA95135 | 0 | 5 |  |  |  |  |
| RIL | 148 | 108 | 1:1 | 3.14 | 1 | 0.076 |

DH = Double haploid; RIL = Recombinant inbred line; df = degree of freedom.

Table S 2. Summary statistics of the genetic linkage map of the CM population

| LG name | Markers | Chr. length (cM) | Ave. spacing (cM) | Max. spacing (cM) |
| --- | --- | --- | --- | --- |
| 1A | 52 | 40.0 | 0.8 | 5.0 |
| 1B | 62 | 90.3 | 1.5 | 23.1 |
| 1D | 32 | 79.6 | 2.6 | 23.8 |
| 2A | 94 | 234.8 | 2.5 | 23.5 |
| 2B | 130 | 130.3 | 1.0 | 17.2 |
| 2D | 42 | 160.0 | 3.9 | 25.5 |
| 3A | 103 | 209.3 | 2.1 | 35.6 |
| 3B | 122 | 175.0 | 1.4 | 21.8 |
| 3D | 16 | 69.0 | 4.6 | 19.0 |
| 4A | 73 | 196.2 | 2.7 | 31.7 |
| 4B | 45 | 87.8 | 2.0 | 25.4 |
| 4D | 4 | 16.9 | 5.6 | 6.9 |
| 5A | 124 | 189.1 | 1.5 | 23.4 |
| 5B | 66 | 143.3 | 2.2 | 24.7 |
| 5D | 12 | 56.6 | 5.1 | 19.8 |
| 6A | 74 | 133.5 | 1.8 | 25.9 |
| 6B | 57 | 142.4 | 2.5 | 26.9 |
| 6D | 20 | 68.6 | 3.6 | 25.5 |
| 7A | 51 | 76.1 | 1.5 | 22.4 |
| 7B | 42 | 74.3 | 1.8 | 17.8 |
| 7D | 28 | 55.0 | 2.0 | 26.1 |
| Overall | 1249 | 2428.0 | 2.5 | 22.4 |

LG=Linkage group; cM = centiMorgans; Chr. = chromosome; Ave. = average; Max. = maximum.

Table S 3. Summary statistics of the genetic linkage map of the LA population

| LG name | Markers | Chr. length (cM) | Ave. spacing (cM) | Max. spacing (cM) |
| --- | --- | --- | --- | --- |
| 1A | 275 | 279.8 | 1.0 | 14.5 |
| 1B.1 | 64 | 52.3 | 0.8 | 9.5 |
| 1B.2 | 21 | 40.3 | 2.0 | 14.1 |
| 1D | 171 | 197.3 | 1.2 | 46.4 |
| 2A | 86 | 103.0 | 1.2 | 13.8 |
| 2B | 415 | 302.3 | 0.7 | 23.3 |
| 2D | 104 | 242.3 | 2.4 | 29.1 |
| 3A | 236 | 278.4 | 1.2 | 28.3 |
| 3B | 404 | 358.7 | 0.9 | 13.9 |
| 3D | 18 | 48.8 | 2.9 | 15.1 |
| 4A.1 | 49 | 129.5 | 2.7 | 19.4 |
| 4A.2 | 48 | 38.3 | 0.8 | 9.6 |
| 4B | 172 | 174.9 | 1.0 | 17.5 |
| 4D | 22 | 80.8 | 3.8 | 15.6 |
| 5A | 209 | 341.9 | 1.6 | 24.5 |
| 5B.2 | 107 | 162.7 | 1.5 | 10.5 |
| 5B.1 | 69 | 40.1 | 0.6 | 2.7 |
| 5D | 23 | 51.7 | 2.3 | 11.6 |
| 6A | 137 | 168.6 | 1.2 | 21.0 |
| 6B | 117 | 187.7 | 1.6 | 31.1 |
| 6D.1 | 26 | 52.3 | 2.1 | 12.9 |
| 6D.2 | 41 | 73.7 | 1.8 | 18.0 |
| 7A.1 | 130 | 127.7 | 1.0 | 12.0 |
| 7A.2 | 172 | 179.7 | 1.1 | 21.0 |
| 7B | 260 | 245.0 | 0.9 | 36.9 |
| 7D | 30 | 150.7 | 5.2 | 25.5 |
| overall | 3406 | 4108.7 | 1.2 | 46.4 |

LG=Linkage group; cM = centiMorgans; Chr. = chromosome; Ave. = average; Max. = maximum.

Table S 4. Annotated genes in the *QSr.nc-6D* region using the Chinese Spring IWGSC RefSeq v1.0 genome assembly.

|  |  |  |  |
| --- | --- | --- | --- |
| Gene name | Start | End | Annotation^a^ |
| TraesCS6D02G012400 | 4,838,653 | 4,840,305 | WAT1-related protein |
| TraesCS6D02G012500 | 4,937,161 | 4,939,465 | Nucleolar RNA binding protein |
| TraesCS6D02G012600 | 5,058,868 | 5,066,947 | Disease resistance protein (TIR-NBS-LRR class) |
| TraesCS6D02G012700 | 5,078,407 | 5,084,396 | Disease resistance protein (TIR-NBS-LRR class) |
| TraesCS6D02G012800 | 5,090,832 | 5,094,788 | F-box family protein |
| TraesCS6D02G012900 | 5,149,646 | 5,153,289 | Disease resistance protein (TIR-NBS-LRR class) |
| TraesCS6D02G013000 | 5,176,529 | 5,183,564 | Disease resistance protein (TIR-NBS-LRR class) |
| TraesCS6D02G013100 | 5,193,788 | 5,198,817 | Disease resistance protein (TIR-NBS-LRR class) |
| TraesCS6D02G013200 | 5,278,714 | 5,279,206 | Glutamyl-tRNA(Gln) amidotransferase subunit A |
| TraesCS6D02G013300 | 5,446,476 | 5,447,036 | DNA topoisomerase |
| TraesCS6D02G013400 | 5,477,924 | 5,481,671 | Receptor-like protein kinase, putative, expressed |
| TraesCS6D02G013500 | 5,493,565 | 5,495,912 | Myb/SANT-like DNA-binding domain protein |
| TraesCS6D02G013600 | 5,562,123 | 5,566,425 | Receptor-like protein kinase, putative, expressed |
| TraesCS6D02G013700 | 5,723,637 | 5,726,189 | Zinc finger BED domain-containing protein DAYSLEEPER |
| TraesCS6D02G013800 | 5,838,587 | 5,839,309 | INO80 complex subunit D |
| TraesCS6D02G013900 | 5,844,543 | 5,845,856 | F-box family protein |
| TraesCS6D02G014000 | 5,909,964 | 5,912,742 | RING/U-box superfamily protein |
| TraesCS6D02G014100 | 5,940,883 | 5,942,499 | Tyrosine decarboxylase |
| TraesCS6D02G014200 | 5,976,319 | 5,979,473 | RNA binding protein |
| TraesCS6D02G014300 | 5,980,828 | 5,982,258 | stress response NST1-like protein |
| TraesCS6D02G014400 | 5,987,202 | 5,987,870 | MADS-box transcription factor 21 |
| TraesCS6D02G014500 | 6,001,437 | 6,003,277 | rRNA N-glycosidase |
| TraesCS6D02G014600 | 6,012,843 | 6,020,283 | MADS-box transcription factor |
| TraesCS6D02G014700 | 6,107,726 | 6,135,028 | Two-component response regulator |
| TraesCS6D02G014800 | 6,156,126 | 6,159,148 | O-acyltransferase WSD1 |
| TraesCS6D02G014900 | 6,254,865 | 6,261,284 | Two-component response regulator |
| TraesCS6D02G015000 | 6,274,077 | 6,276,905 | Cytochrome P450 |
| TraesCS6D02G015100 | 6,314,769 | 6,316,761 | 4,5-dioxygenase-like protein |
| TraesCS6D02G015200 | 6,319,338 | 6,321,386 | Flavonoid 3'-hydroxylase |
| TraesCS6D02G015300 | 6,328,264 | 6,328,920 | Flavonoid 3'-hydroxylase |
| TraesCS6D02G015400 | 6,342,582 | 6,344,284 | Isoflavone reductase-like protein |
| TraesCS6D02G015500 | 6,351,577 | 6,353,794 | Leucine-rich repeat receptor-like protein kinase family protein |
| TraesCS6D02G015600 | 6,354,023 | 6,354,982 | Leucine-rich repeat receptor-like protein kinase family protein |
| TraesCS6D02G015700 | 6,370,264 | 6,373,164 | Leucine-rich repeat receptor-like protein kinase family protein |
|  |  |  |  |

^a^Annotated disease resistance proteins in the *QSr.nc-6D* region correspond to toll/interleukin-1 receptor transmembrane domain (TIR), nucleotide-binding sites (NBS), and leucine-rich repeat (LRR) class.

Table S 5. Validation of co-segregating *SrA2K* KASP markers for marker-assisted selection purposes in the Soft Red Winter Wheat. Lines were screened at seedling stage using *Pgt* raceTTKSK s in 2022.

|  |  |  |  |  |  |  |
| --- | --- | --- | --- | --- | --- | --- |
| Line | TTKSK reaction/  Locus | kwm112 | kwm918 | IWB30767_12 | NCB_6D_5420176 | *Sr24* |
|  |  | 6D_5,064,580 | 6D_5,150,751 | 6D_5,194,788 | 6D_5,420,176 | 3D_624129157 |
| AGS2000 | R/*SrA2K* | T | T | T | T | C |
| P2128 | R/*SrA2K* | T | T | T | T | C |
| FL16009LDH-16 | R/*SrA2K* | T | T | T | T | C |
| FL16045LDH-25 | R/*SrA2K* | T | T | T | T | C |
| TN 2203 | R/*SrA2K* | T | T | T | T | C |
| 17VDH-SR203-143 | R/*SrA2K* | T | T | T | T | C |
| LA14234CBW-31 | R/*SrA2K* | T | T | T | T | C |
| GA161137LDH-23-20LE3 | R/*Sr24* | T | T | T | T | T |
| LA15093SB-30-2 | R/*Sr24* | T | T | T | T | T |
| TWR 19016 | R/*Sr24* | H | H | H | C | T |
| TWR 09056 | R/*Sr24* | H | H | H | C | T |
| VA19FHB-05 | R/*Sr24* | H | H | H | C | T |
| P2143 | R/*Sr36* | H | H | H | C | C |
| MI20R0103 | R/*Sr1RS^Amigo^* | H | H | H | C | C |
| FL15105-LDH039 | R/*Sr36* | H | H | H | C | C |
| 15VTK-1-101 | R/*Sr36* | H | H | H | C | C |
| KWS347 | R | H | H | H | C | C |
| KWS405 | R | H | H | H | C | C |
| Branson | S | H | C | H | C | C |
| MO080104 | S | H | H | H | C | C |
| Hilliard | S | H | H | H | C | C |
| Pioneer Brand 25R46 | S | H | H | H | C | C |
| 17NSVX310257 | S | H | H | H | C | C |
| 17NSVZ311562 | S | H | H | H | C | C |
| NS18VW311562 | S | C | C | H | C | C |
| IL15-23972 | S | C | C | H | C | C |
| IL17-17739 | S | H | C | H | C | C |
| IL17-23874 | S | C | C | H | C | C |
| KWS398 | S | H | H | H | C | C |
| KWS403 | S | H | H | H | C | C |
| KWS414 | S | H | H | H | C | C |
| TWR 19009 | S | H | C | H | C | C |
| X12-3010-4-4-1 | S | C | H | H | C | C |
| X11-0039-1-17-5 | S | H | H | H | C | C |
| VA19W-29 | S | H | H | H | C | C |
| VA19FHB-22 | S | H | H | H | C | C |
| 16VDH-SRW03_018 | S | H | H | H | C | C |
| MI18R0194 | S | H | H | H | C | C |
| MI19R0003 | S | H | H | H | C | C |
| P2104 | S | H | H | H | C | C |
| UMD-21-4 | S | C | C | H | C | C |
| UMD-21-5 | S | C | C | H | C | C |
| UMD-21-6 | S | C | C | H | C | C |
| Jamestown | S | H | H | H | C | C |
| Pioneer Brand 26R41 | S | H | H | H | C | C |
| NC15V25-20 | S | H | H | H | C | C |
| NC13955-G125 | S | H | H | H | C | C |
| NC18-16920 | S | H | H | H | C | C |
| TN 2202 | S | H | H | H | C | C |
| GA131218-1-2-7-20E15 | S | H | H | H | C | C |
| GA151313-LDH-192-20E48 | S | H | H | H | C | C |
| GA161240LDH-113-20LE6 | S | H | H | H | C | C |
| SS18JL502143N | S | H | H | H | C | C |
| SS18JL502282N | S | H | H | H | C | C |
| TX18D3212 | S | H | H | H | C | C |
| VA19W-31 | S | H | H | H | C | C |
| KWS419 | S | H | H | H | C | C |
| LA13154D-WN1 | S | H | H | H | C | C |
| LA14152SB-BR52-3 | S | H | H | H | C | C |
| UMD-21-1 | S | C | C | H | C | C |
| UMD-21-2 | S | C | C | H | C | C |
| UMD-21-3 | S | H | H | H | C | C |
| X11-0120-12-4-3 | S | H | C | C | C | C |
| TN 2201 | S | C | C | C | C | C |
| TX17D2337 | S | C | C | C | C | C |
| TWR 19003 | S | C | C | C | C | C |
| TWR 19005 | S | C | C | C | C | C |
| TWR 19008 | S | C | C | C | C | C |
|  |  |  |  |  |  |  |

AGS2000 is the positive control for the *SrA2K*-resistant haplotype in hexaploid wheat; R=resistant and S=susceptible; kwm are markers reported by Kassa et al. (2016); IWB is a 90K SNP-based KASP assay; NCB corresponds to exome capture KASP assay; *Sr24* KASP assay was developed at the Eastern Regional Small Grains Genotyping Laboratory (ERSGGL) using the 10+Genome LongReach Lancer Seq v1.0; 6D and 3D marker genomic positions (bp) are based on Chinese Spring IWGSC RefSeq v1.0 genome assembly; T indicates resistant allele; C indicates susceptible allele; N indicates no amplification; H indicates heterozygous

Table S 6. – Identified annotated NLR gene clusters in hexaploid wheat genomes collinear to *SrA2K* region using the TGT tools (Chen et al., 2020). In bold are indicated NLR clusters.

|  |  |  |
| --- | --- | --- |
| Gene | Location | Description |
| Chinese Spring: |  |  |
| **TraesCS6D02G012600** | **5058868-5066947(+)** | **Disease resistance protein RGA5** |
| **TraesCS6D02G012700** | **5078407-5084396(+)** | **Disease resistance protein RGA5** |
| TraesCS6D02G012800 | 5090832-5094788(-) | F-box family protein |
| **TraesCS6D02G012900** | **5149646-5153289(-)** | **Disease resistance protein RGA5** |
| **TraesCS6D02G013000** | **5176529-5183564(-)** | **Disease resistance protein RGA5** |
| **TraesCS6D02G013100** | **5193788-5198817(-)** | **Disease resistance protein RGA5** |
| TraesCS6D02G013200 | 5278714-5279206(-) | Glutamyl-tRNA(Gln) amidotransferase subunit A |
| TraesCS6D02G013300 | 5446476-5447036(-) | DNA topoisomerase |
| TraesCS6D02G013400 | 5477924-5481671(-) | Cysteine-rich receptor-like protein kinase 6 |
| TraesCS6D02G013500 | 5493565-5495912(+) | Myb/SANT-like DNA-binding domain protein |
| TraesCS6D02G013600 | 5562123-5566425(+) | Cysteine-rich receptor-like protein kinase 6 |
| TraesCS6D02G013700 | 5723637-5726189(+) | Zinc finger BED domain-containing protein DAYSLEEPER |
| TraesCS6D02G013800 | 5838587-5839309(+) | INO80 complex subunit D |
| TraesCS6D02G013900 | 5844543-5845856(-) | F-box family protein |
| TraesCS6D02G014000 | 5909964-5912742(-) | RING/U-box superfamily protein |
| TraesCS6D02G014100 | 5940883-5942499(-) | Tyrosine/DOPA decarboxylase 3 |
| TraesCS6D02G014200 | 5976319-5979473(-) | MKI67 FHA domain-interacting nucleolar phosphoprotein |
| TraesCS6D02G014300 | 5980828-5982258(-) | stress response NST1-like protein |
| TraesCS6D02G014400 | 5987202-5987870(-) | MADS-box transcription factor 21 |
| TraesCS6D02G014500 | 6001437-6003277(-) | Ribosome-inactivating protein 9 |
| TraesCS6D02G014600 | 6012843-6020283(+) | MADS-box transcription factor 56 |
| TraesCS6D02G014700 | 6107726-6135028(-) | Two-component response regulator ORR21 |
| TraesCS6D02G014800 | 6156126-6159148(-) | O-acyltransferase WSD1 |
| TraesCS6D02G014900 | 6254865-6261284(-) | Two-component response regulator |
| TraesCS6D02G015000 | 6274077-6276905(+) | Cytochrome P450 709B2 |
| TraesCS6D02G015100 | 6314769-6316761(-) | Extradiol ring-cleavage dioxygenase |
| TraesCS6D02G015200 | 6319338-6321386(+) | Flavonoid 3'-monooxygenase CYP75B4 |
| TraesCS6D02G015300 | 6328264-6328920(+) | Flavonoid 3'-monooxygenase CYP75B4 |
| TraesCS6D02G015400 | 6342582-6344284(-) | Isoflavone reductase homolog IRL |
| TraesCS6D02G015500 | 6351577-6353794(+) | LRR receptor-like serine/threonine-protein kinase FLS2 |
| TraesCS6D02G015600 | 6354023-6354982(+) | Receptor-like protein EIX2 |
| TraesCS6D02G015700 | 6370264-6373164(+) | Receptor-like protein EIX2 |
| Fielder: |  |  |
| **TraesFLD6D01G004600** | **1441324-1448569(+)** | **Disease resistance protein RGA5** |
| **TraesFLD6D01G004700** | **1461323-1465021(+)** | **Disease resistance protein RGA5** |
| TraesFLD6D01G004800 | 1473025-1476754(-) | NA |
| **TraesFLD6D01G004900** | **1531278-1534980(-)** | **Disease resistance protein RGA5** |
| **TraesFLD6D01G005000** | **1564025-1564872(-)** | **Disease resistance protein PIK6-NP** |
| **TraesFLD6D01G005100** | **1575180-1579505(-)** | **Disease resistance protein RGA5** |
| **TraesFLD6D01G005200** | **1603193-1609397(-)** | **Disease resistance protein RGA5** |
| **TraesFLD6D01G005300** | **1620050-1653909(-)** | **Disease resistance protein RGA5** |
| **TraesFLD6D01G005400** | **1692091-1698434(-)** | **Disease resistance protein RGA5** |
| **TraesFLD6D01G005500** | **1708742-1713066(-)** | **Disease resistance protein RGA5** |
| TraesFLD6D01G005600 | 1787898-1788390(-) | NA |
| TraesFLD6D01G005700 | 1953796-1954356(-) | NA |
| TraesFLD6D01G005800 | 1985267-1988678(-) | Cysteine-rich receptor-like protein kinase 6 |
| TraesFLD6D01G005900 | 2001311-2002722(+) | NA |
| TraesFLD6D01G006000 | 2068289-2072321(+) | Cysteine-rich receptor-like protein kinase 6 |
| TraesFLD6D01G006100 | 2482817-2485028(+) | NA |
| TraesFLD6D01G006200 | 2587375-2588448(-) | NA |
| TraesFLD6D01G006300 | 2596196-2596918(+) | NA |
| TraesFLD6D01G006400 | 2602162-2603448(-) | NA |
| TraesFLD6D01G006500 | 2666216-2668994(-) | NA |
| TraesFLD6D01G006600 | 2696946-2698424(-) | Tyrosine/DOPA decarboxylase 3 |
| TraesFLD6D01G006700 | 2852496-2854528(-) | MKI67 FHA domain-interacting nucleolar phosphoprotein |
| TraesFLD6D01G006800 | 2857105-2857650(-) | NA |
| TraesFLD6D01G006900 | 2863167-2863835(-) | NA |
| TraesFLD6D01G007000 | 2878238-2879014(-) | Ribosome-inactivating protein 9 |
| TraesFLD6D01G007100 | 2888647-2895391(+) | MADS-box transcription factor 56 |
| TraesFLD6D01G007200 | 2983206-3008928(-) | Two-component response regulator ORR21 |
| TraesFLD6D01G007300 | 3029115-3032005(-) | O-acyltransferase WSD1 |
| TraesFLD6D01G007400 | 3120660-3121298(-) | NA |
| TraesFLD6D01G007500 | 3126924-3133343(-) | Two-component response regulator |
| TraesFLD6D01G007600 | 3145754-3148073(+) | Cytochrome P450 709B2 |
| TraesFLD6D01G007700 | 3186578-3187802(-) | Extradiol ring-cleavage dioxygenase |
| TraesFLD6D01G007800 | 3190486-3192292(+) | Flavonoid 3'-monooxygenase CYP75B4 |
| TraesFLD6D01G007900 | 3199329-3199985(+) | Flavonoid 3'-monooxygenase CYP75B4 |
| TraesFLD6D01G008000 | 3213899-3215200(-) | Isoflavone reductase homolog IRL |
| TraesFLD6D01G008100 | 3220639-3222660(+) | LRR receptor-like serine/threonine-protein kinase FLS2 |
| TraesFLD6D01G008200 | 3223108-3223848(+) | Receptor-like protein EIX2 |
| TraesFLD6D01G008300 | 3239349-3242249(+) | Receptor-like protein EIX2 |
| Julius: |  |  |
| TraesJUL6D01G030100 | 11985958-11986450(-) | NA |
| TraesJUL6D01G030300 | 12149314-12149874(-) | NA |
| TraesJUL6D01G030400 | 12184493-12187904(-) | Cysteine-rich receptor-like protein kinase 6 |
| TraesJUL6D01G030500 | 12200537-12201948(+) | NA |
| TraesJUL6D01G030600 | 12266917-12270949(+) | Cysteine-rich receptor-like protein kinase 6 |
| TraesJUL6D01G030700 | 12646448-12648780(+) | NA |
| TraesJUL6D01G030900 | 12759160-12759882(+) | NA |
| TraesJUL6D01G031000 | 12765226-12766512(-) | NA |
| TraesJUL6D01G031100 | 12829035-12831813(-) | NA |
| TraesJUL6D01G031200 | 12859080-12860558(-) | Tyrosine/DOPA decarboxylase 3 |
| TraesJUL6D01G031300 | 12916470-12918502(-) | MKI67 FHA domain-interacting nucleolar phosphoprotein |
| TraesJUL6D01G031400 | 12921079-12921624(-) | NA |
| TraesJUL6D01G031500 | 12927141-12927809(-) | NA |
| TraesJUL6D01G031600 | 12942402-12943178(-) | Ribosome-inactivating protein 9 |
| TraesJUL6D01G031700 | 12953018-12959763(+) | MADS-box transcription factor 56 |
| TraesJUL6D01G031800 | 12953018-12959763(+) | MADS-box transcription factor 56 |
| TraesJUL6D01G031900 | 13053527-13079535(-) | Two-component response regulator ORR21 |
| TraesJUL6D01G032000 | 13099916-13102806(-) | O-acyltransferase WSD1 |
| TraesJUL6D01G032100 | 13197450-13203869(-) | Two-component response regulator |
| TraesJUL6D01G032200 | 13216077-13218396(+) | Cytochrome P450 709B2 |
| TraesJUL6D01G032300 | 13255977-13257201(-) | Extradiol ring-cleavage dioxygenase |
| TraesJUL6D01G032400 | 13259885-13261691(+) | Flavonoid 3'-monooxygenase CYP75B4 |
| TraesJUL6D01G032500 | 13268791-13269447(+) | Flavonoid 3'-monooxygenase CYP75B4 |
| TraesJUL6D01G032600 | 13283359-13284660(-) | Isoflavone reductase homolog IRL |
| TraesJUL6D01G032700 | 13291590-13293611(+) | LRR receptor-like serine/threonine-protein kinase FLS2 |
| TraesJUL6D01G032800 | 13294059-13294799(+) | Receptor-like protein EIX2 |
| TraesJUL6D01G032900 | 13310300-13313200(+) | Receptor-like protein EIX2 |
| TraesJUL6D01G033000 | 13365303-13367030(+) | Receptor-like protein EIX2 |
| TraesJUL6D01G033100 | 13367088-13368197(+) | Receptor-like protein EIX2 |
| TraesJUL6D01G033200 | 13380890-13382752(+) | Probable inactive leucine-rich repeat receptor kinase XIAO |
| TraesJUL6D01G033400 | 13435276-13437900(+) | Receptor-like protein EIX2 |
| TraesJUL6D01G033500 | 13510124-13512971(-) | Trihelix transcription factor DF1 |
| TraesJUL6D01G033600 | 13765769-13767956(+) | NA |
| TraesJUL6D01G033700 | 13912284-13918663(-) | NA |
| TraesJUL6D01G033800 | 13925414-13928143(-) | Receptor-like protein 43 |
| TraesJUL6D01G033900 | 13930240-13931545(+) | Probable glutathione S-transferase GSTF1 |
| TraesJUL6D01G034000 | 13980118-13982808(+) | Receptor-like protein EIX2 |
| TraesJUL6D01G034100 | 14033219-14036708(+) | Wall-associated receptor kinase 5 |
| TraesJUL6D01G034200 | 14033219-14036714(+) | Wall-associated receptor kinase 5 |
| TraesJUL6D01G034300 | 14042614-14045109(+) | Putative nitric oxide synthase |
| TraesJUL6D01G034400 | 14047260-14054857(+) | Ubiquitin-like domain-containing protein CIP73 |
| TraesJUL6D01G034500 | 14056062-14056433(-) | NA |
| TraesJUL6D01G034600 | 14056062-14056445(-) | Outer envelope protein 61 |
| TraesJUL6D01G034700 | 14104192-14104602(-) | NA |
| TraesJUL6D01G034800 | 14111019-14111471(+) | NA |
| TraesJUL6D01G034900 | 14155022-14157283(-) | BURP domain-containing protein 14 |
| **TraesJUL6D01G035000** | **14225397-14229431(-)** | **Disease resistance protein RGA5** |
| **TraesJUL6D01G035100** | **14234987-14237371(-)** | **Disease resistance protein RGA5** |
| TraesJUL6D01G035200 | 14341952-14345678(+) | NA |
| **TraesJUL6D01G035300** | **14353968-14357666(-)** | **Disease resistance protein RGA5** |
| **TraesJUL6D01G035400** | **14370501-14377734(-)** | **Disease resistance protein RGA5** |
| Jagger: |  |  |
| **TraesJAG6D01G013100** | **4479675-4486908(+)** | **Disease resistance protein RGA5** |
| **TraesJAG6D01G013200** | **4499686-4503384(+)** | **Disease resistance protein RGA5** |
| TraesJAG6D01G013300 | 4511404-4515208(-) | NA |
| TraesJAG6D01G013400 | 4586551-4588029(+) | Tyrosine/DOPA decarboxylase 3 |
| TraesJAG6D01G013500 | 4615298-4618076(+) | NA |
| TraesJAG6D01G013600 | 4681078-4682364(+) | NA |
| TraesJAG6D01G013700 | 4687600-4688322(-) | NA |
| TraesJAG6D01G013900 | 4800570-4802902(-) | NA |
| TraesJAG6D01G014000 | 5161399-5165431(-) | Cysteine-rich receptor-like protein kinase 6 |
| TraesJAG6D01G014100 | 5231766-5233177(-) | NA |
| TraesJAG6D01G014200 | 5245810-5249221(+) | Cysteine-rich receptor-like protein kinase 6 |
| TraesJAG6D01G014300 | 5287685-5288245(+) | NA |
| TraesJAG6D01G014500 | 5452869-5453361(+) | NA |
| **TraesJAG6D01G014600** | **5534196-5538013(+)** | **Disease resistance protein RGA5** |
| TraesJAG6D01G014700 | 5554058-5556090(-) | MKI67 FHA domain-interacting nucleolar phosphoprotein |
| TraesJAG6D01G014800 | 5558667-5559212(-) | NA |
| TraesJAG6D01G014900 | 5564729-5565397(-) | NA |
| TraesJAG6D01G015000 | 5580501-5581277(-) | Ribosome-inactivating protein 9 |
| TraesJAG6D01G015100 | 5591327-5598072(+) | MADS-box transcription factor 56 |
| TraesJAG6D01G015200 | 5686934-5712628(-) | Two-component response regulator ORR21 |
| TraesJAG6D01G015300 | 5733184-5736074(-) | O-acyltransferase WSD1 |
| TraesJAG6D01G015400 | 5826106-5826744(-) | NA |
| TraesJAG6D01G015500 | 5832370-5838789(-) | Two-component response regulator |
| TraesJAG6D01G015600 | 5851511-5853830(+) | Cytochrome P450 709B2 |
| TraesJAG6D01G015700 | 5892979-5894203(-) | Extradiol ring-cleavage dioxygenase |
| TraesJAG6D01G015800 | 5896887-5898693(+) | Flavonoid 3'-monooxygenase CYP75B4 |
| TraesJAG6D01G015900 | 5905925-5906581(+) | Flavonoid 3'-monooxygenase CYP75B4 |
| TraesJAG6D01G016000 | 5920493-5921794(-) | Isoflavone reductase homolog IRL |
| TraesJAG6D01G016100 | 5928750-5930771(+) | LRR receptor-like serine/threonine-protein kinase FLS2 |
| TraesJAG6D01G016200 | 5931219-5931959(+) | Receptor-like protein EIX2 |
| TraesJAG6D01G016300 | 5947460-5950360(+) | Receptor-like protein EIX2 |
| Arina LrFor: |  |  |
| **TraesARI6D01G014400** | **5202671-5209916(+)** | **Disease resistance protein RGA5** |
| **TraesARI6D01G014500** | **5222708-5226349(+)** | **Disease resistance protein RGA5** |
| TraesARI6D01G014600 | 5234350-5238075(-) | NA |
| **TraesARI6D01G014700** | **5292763-5296483(-)** | **Disease resistance protein RGA5** |
| **TraesARI6D01G014800** | **5320033-5326376(-)** | **Disease resistance protein RGA5** |
| **TraesARI6D01G014900** | **5336841-5341159(-)** | **Disease resistance protein RGA5** |
| TraesARI6D01G015000 | 5416636-5417128(-) | NA |
| TraesARI6D01G015200 | 5582125-5582685(-) | NA |
| TraesARI6D01G015300 | 5613072-5616483(-) | Cysteine-rich receptor-like protein kinase 6 |
| TraesARI6D01G015400 | 5629117-5630528(+) | NA |
| TraesARI6D01G015500 | 5696118-5700150(+) | Cysteine-rich receptor-like protein kinase 6 |
| TraesARI6D01G015600 | 6073228-6075560(+) | NA |
| TraesARI6D01G015700 | 6177832-6178905(-) | NA |
| TraesARI6D01G015800 | 6186857-6187579(+) | NA |
| TraesARI6D01G015900 | 6192815-6194101(-) | NA |
| TraesARI6D01G016000 | 6256718-6259496(-) | NA |
| TraesARI6D01G016100 | 6287453-6288931(-) | Tyrosine/DOPA decarboxylase 3 |
| TraesARI6D01G016200 | 6346533-6348565(-) | MKI67 FHA domain-interacting nucleolar phosphoprotein |
| TraesARI6D01G016300 | 6351142-6351687(-) | NA |
| TraesARI6D01G016400 | 6357204-6357872(-) | NA |
| TraesARI6D01G016500 | 6372372-6373148(-) | Ribosome-inactivating protein 9 |
| TraesARI6D01G016600 | 6382834-6389645(+) | MADS-box transcription factor 56 |
| TraesARI6D01G016700 | 6483867-6518208(-) | Two-component response regulator ORR21 |
| TraesARI6D01G016800 | 6538491-6541377(-) | O-acyltransferase WSD1 |
| TraesARI6D01G016900 | 6621843-6622478(-) | NA |
| TraesARI6D01G017000 | 6628134-6634575(-) | Two-component response regulator |
| TraesARI6D01G017100 | 6657846-6660176(+) | Cytochrome P450 709B2 |
| TraesARI6D01G017200 | 6698712-6699776(-) | Extradiol ring-cleavage dioxygenase |
| TraesARI6D01G017300 | 6702696-6704507(+) | Flavonoid 3'-monooxygenase CYP75B4 |
| TraesARI6D01G017400 | 6702696-6704507(+) | Flavonoid 3'-monooxygenase CYP75B4 |
| TraesARI6D01G017500 | 6720095-6721395(-) | Isoflavone reductase homolog IRL |
| TraesARI6D01G017600 | 6743323-6743793(+) | Receptor-like protein EIX2 |
| TraesARI6D01G017700 | 6820302-6822029(+) | Receptor-like protein EIX2 |
| TraesARI6D01G017800 | 6820302-6839184(+) | Receptor-like protein EIX2 |
| TraesARI6D01G017900 | 6822087-6823196(+) | Receptor-like protein EIX2 |
| TraesARI6D01G018100 | 6838444-6839184(+) | Receptor-like protein EIX2 |
| TraesARI6D01G018200 | 6857566-6858755(+) | Receptor-like protein EIX2 |
| TraesARI6D01G018300 | 6857566-6860188(+) | Receptor-like protein EIX2 |
| CDC Stanley: |  |  |
| TraesSTA6D01G002800 | 970016-972638(-) | Receptor-like protein EIX2 |
| TraesSTA6D01G002900 | 991329-1010951(-) | Receptor-like protein EIX2 |
| TraesSTA6D01G003000 | 1009224-1010951(-) | Receptor-like protein EIX2 |
| TraesSTA6D01G003200 | 1110652-1111952(+) | Isoflavone reductase homolog IRL |
| TraesSTA6D01G003300 | 1127792-1129603(-) | Flavonoid 3'-monooxygenase CYP75B4 |
| TraesSTA6D01G003400 | 1127792-1129603(-) | Flavonoid 3'-monooxygenase CYP75B4 |
| TraesSTA6D01G003500 | 1132523-1133587(+) | Extradiol ring-cleavage dioxygenase |
| TraesSTA6D01G003600 | 1172145-1174475(-) | Cytochrome P450 709B2 |
| TraesSTA6D01G003700 | 1198206-1204647(+) | Two-component response regulator |
| TraesSTA6D01G003800 | 1210303-1210938(+) | NA |
| TraesSTA6D01G003900 | 1292348-1295234(+) | O-acyltransferase WSD1 |
| TraesSTA6D01G004000 | 1316519-1351032(+) | Two-component response regulator ORR21 |
| TraesSTA6D01G004100 | 1451522-1458255(-) | MADS-box transcription factor 56 |
| TraesSTA6D01G004200 | 1468383-1469159(+) | Ribosome-inactivating protein 9 |
| TraesSTA6D01G004300 | 1483923-1484591(+) | NA |
| TraesSTA6D01G004400 | 1490108-1490653(+) | NA |
| TraesSTA6D01G004500 | 1493230-1495262(+) | MKI67 FHA domain-interacting nucleolar phosphoprotein |
| TraesSTA6D01G004600 | 1603448-1606226(+) | NA |
| TraesSTA6D01G004700 | 1652956-1654687(+) | NA |
| TraesSTA6D01G004800 | 1678387-1679673(+) | NA |
| TraesSTA6D01G004900 | 1684885-1685607(-) | NA |
| TraesSTA6D01G005200 | 1797281-1799613(-) | NA |
| TraesSTA6D01G005300 | 2148305-2152337(-) | Cysteine-rich receptor-like protein kinase 6 |
| TraesSTA6D01G005400 | 2224168-2225579(-) | NA |
| TraesSTA6D01G005500 | 2238213-2241624(+) | Cysteine-rich receptor-like protein kinase 6 |
| TraesSTA6D01G005600 | 2272278-2272838(+) | NA |
| TraesSTA6D01G005800 | 2439442-2439934(+) | NA |
| **TraesSTA6D01G005900** | **2516663-2521172(+)** | **Disease resistance protein RGA5** |
| **TraesSTA6D01G006000** | **2531582-2537587(+)** | **Disease resistance protein RGA5** |
| **TraesSTA6D01G006100** | **2531582-2537786(+)** | **Disease resistance protein RGA5** |
| **TraesSTA6D01G006200** | **2531582-2537925(+)** | **Disease resistance protein RGA5** |
| **TraesSTA6D01G006300** | **2561495-2565473(+)** | **Disease resistance protein RGA5** |
| TraesSTA6D01G006400 | 2621190-2625022(+) | NA |
| **TraesSTA6D01G006500** | **2633244-2636942(-)** | **Disease resistance protein RGA5** |
| **TraesSTA6D01G006600** | **2649935-2657079(-)** | **Disease resistance protein RGA5** |
| LongReach Lancer: |  |  |
| TraesLAC6D01G018600 | 7012312-7015212(-) | Receptor-like protein EIX2 |
| TraesLAC6D01G018700 | 7030572-7031312(-) | Receptor-like protein EIX2 |
| TraesLAC6D01G018800 | 7031760-7033781(-) | LRR receptor-like serine/threonine-protein kinase FLS2 |
| TraesLAC6D01G018900 | 7040678-7041979(+) | Isoflavone reductase homolog IRL |
| TraesLAC6D01G019000 | 7055641-7056297(-) | Flavonoid 3'-monooxygenase CYP75B4 |
| TraesLAC6D01G019100 | 7063334-7065140(-) | Flavonoid 3'-monooxygenase CYP75B4 |
| TraesLAC6D01G019200 | 7067824-7069048(+) | Extradiol ring-cleavage dioxygenase |
| TraesLAC6D01G019300 | 7107798-7110117(-) | Cytochrome P450 709B2 |
| TraesLAC6D01G019400 | 7122730-7129149(+) | Two-component response regulator |
| TraesLAC6D01G019500 | 7134775-7135413(+) | NA |
| TraesLAC6D01G019600 | 7223624-7226514(+) | O-acyltransferase WSD1 |
| TraesLAC6D01G019700 | 7246796-7271184(+) | Two-component response regulator ORR21 |
| TraesLAC6D01G019800 | 7358829-7365645(-) | MADS-box transcription factor 56 |
| TraesLAC6D01G019900 | 7375329-7376105(+) | Ribosome-inactivating protein 9 |
| TraesLAC6D01G020000 | 7390585-7391253(+) | NA |
| TraesLAC6D01G020100 | 7396770-7397315(+) | NA |
| TraesLAC6D01G020200 | 7399892-7401924(+) | MKI67 FHA domain-interacting nucleolar phosphoprotein |
| TraesLAC6D01G020300 | 7451243-7452721(+) | Tyrosine/DOPA decarboxylase 3 |
| TraesLAC6D01G020400 | 7480720-7483498(+) | NA |
| TraesLAC6D01G020500 | 7545308-7546594(+) | NA |
| TraesLAC6D01G020600 | 7551824-7552546(-) | NA |
| TraesLAC6D01G020800 | 7663922-7666254(-) | NA |
| TraesLAC6D01G020900 | 8034863-8038895(-) | Cysteine-rich receptor-like protein kinase 6 |
| TraesLAC6D01G021000 | 8104594-8106005(-) | NA |
| TraesLAC6D01G021100 | 8118639-8122050(+) | Cysteine-rich receptor-like protein kinase 6 |
| TraesLAC6D01G021200 | 8152071-8152631(+) | NA |
| TraesLAC6D01G021400 | 8313635-8314127(+) | NA |
| **TraesLAC6D01G021500** | **8389286-8393770(+)** | **Disease resistance protein RGA5** |
| **TraesLAC6D01G021600** | **8404080-8438265(+)** | **Disease resistance protein RGA5** |
| **TraesLAC6D01G021700** | **8404101-8404927(+)** | **Disease resistance protein PIK6-NP** |
| TraesLAC6D01G021800 | 8492893-8496618(+) | NA |
| **TraesLAC6D01G021900** | **8504704-8508402(-)** | **Disease resistance protein RGA5** |
| **TraesLAC6D01G022000** | **8521019-8528377(-)** | **Disease resistance protein RGA5** |
| **TraesLAC6D01G022100** | **8521127-8528377(-)** | **Disease resistance protein RGA5** |
| Mace: |  |  |
| **TraesMAC6D01G014200** | **5257206-5264439(+)** | **Disease resistance protein RGA5** |
| **TraesMAC6D01G014300** | **5277150-5280791(+)** | **Disease resistance protein RGA5** |
| TraesMAC6D01G014400 | 5288792-5292517(-) | NA |
| **TraesMAC6D01G014500** | **5347303-5351159(-)** | **Disease resistance protein RGA5** |
| **TraesMAC6D01G014600** | **5374708-5381051(-)** | **Disease resistance protein RGA5** |
| **TraesMAC6D01G014700** | **5391337-5395891(-)** | **Disease resistance protein RGA5** |
| TraesMAC6D01G014800 | 5471180-5471672(-) | NA |
| TraesMAC6D01G015000 | 5633820-5634380(-) | NA |
| TraesMAC6D01G015100 | 5665353-5668764(-) | Cysteine-rich receptor-like protein kinase 6 |
| TraesMAC6D01G015200 | 5681398-5682809(+) | NA |
| TraesMAC6D01G015300 | 5748094-5752126(+) | Cysteine-rich receptor-like protein kinase 6 |
| TraesMAC6D01G015400 | 6125148-6127480(+) | NA |
| TraesMAC6D01G015600 | 6238791-6239513(+) | NA |
| TraesMAC6D01G015700 | 6244739-6246025(-) | NA |
| TraesMAC6D01G015800 | 6308936-6311714(-) | NA |
| TraesMAC6D01G015900 | 6339733-6341211(-) | Tyrosine/DOPA decarboxylase 3 |
| TraesMAC6D01G016000 | 6381545-6383577(-) | MKI67 FHA domain-interacting nucleolar phosphoprotein |
| TraesMAC6D01G016100 | 6386154-6386699(-) | NA |
| TraesMAC6D01G016200 | 6392216-6392884(-) | NA |
| TraesMAC6D01G016300 | 6407372-6408148(-) | Ribosome-inactivating protein 9 |
| TraesMAC6D01G016400 | 6417854-6424623(+) | MADS-box transcription factor 56 |
| TraesMAC6D01G016500 | 6518431-6552363(-) | Two-component response regulator ORR21 |
| TraesMAC6D01G016600 | 6572759-6575645(-) | O-acyltransferase WSD1 |
| TraesMAC6D01G016700 | 6655718-6656353(-) | NA |
| TraesMAC6D01G016800 | 6662009-6668450(-) | Two-component response regulator |
| TraesMAC6D01G016900 | 6692441-6694771(+) | Cytochrome P450 709B2 |
| TraesMAC6D01G017000 | 6733712-6734776(-) | Extradiol ring-cleavage dioxygenase |
| TraesMAC6D01G017100 | 6737696-6739507(+) | Flavonoid 3'-monooxygenase CYP75B4 |
| TraesMAC6D01G017200 | 6755102-6756402(-) | Isoflavone reductase homolog IRL |
| TraesMAC6D01G017300 | 6856581-6858306(+) | Receptor-like protein EIX2 |
| TraesMAC6D01G017400 | 6858366-6859475(+) | Receptor-like protein EIX2 |
| TraesMAC6D01G017600 | 6875043-6875783(+) | Receptor-like protein EIX2 |
| TraesMAC6D01G017700 | 6894146-6897681(+) | Receptor-like protein EIX2 |
| Norin61: |  |  |
| **TraesNOR6D01G013800** | **4863693-4870944(+)** | **Disease resistance protein RGA5** |
| **TraesNOR6D01G013900** | **4883385-4887083(+)** | **Disease resistance protein RGA5** |
| TraesNOR6D01G014000 | 4895193-4898923(-) | NA |
| **TraesNOR6D01G014100** | **4955090-4958771(-)** | **Disease resistance protein RGA5** |
| **TraesNOR6D01G014200** | **4982495-4988838(-)** | **Disease resistance protein RGA5** |
| **TraesNOR6D01G014300** | **4999194-5003552(-)** | **Disease resistance protein RGA5** |
| TraesNOR6D01G014400 | 5078590-5079082(-) | NA |
| TraesNOR6D01G014600 | 5242123-5242683(-) | NA |
| TraesNOR6D01G014700 | 5273394-5276805(-) | Cysteine-rich receptor-like protein kinase 6 |
| TraesNOR6D01G014800 | 5289438-5290849(+) | NA |
| TraesNOR6D01G014900 | 5356404-5360436(+) | Cysteine-rich receptor-like protein kinase 6 |
| TraesNOR6D01G015000 | 5737784-5740116(+) | NA |
| TraesNOR6D01G015200 | 5842601-5843662(-) | NA |
| TraesNOR6D01G015300 | 5851439-5852160(+) | NA |
| TraesNOR6D01G015400 | 5857394-5858680(-) | NA |
| TraesNOR6D01G015500 | 5920969-5923747(-) | NA |
| TraesNOR6D01G015600 | 5951783-5953261(-) | Tyrosine/DOPA decarboxylase 3 |
| TraesNOR6D01G015700 | 6002720-6004752(-) | MKI67 FHA domain-interacting nucleolar phosphoprotein |
| TraesNOR6D01G015800 | 6007329-6007874(-) | NA |
| TraesNOR6D01G015900 | 6013391-6014059(-) | NA |
| TraesNOR6D01G016000 | 6028575-6029351(-) | Ribosome-inactivating protein 9 |
| TraesNOR6D01G016100 | 6039077-6045891(+) | MADS-box transcription factor 56 |
| TraesNOR6D01G016200 | 6133444-6159236(-) | Two-component response regulator ORR21 |
| TraesNOR6D01G016300 | 6179371-6182261(-) | O-acyltransferase WSD1 |
| TraesNOR6D01G016400 | 6270262-6270900(-) | NA |
| TraesNOR6D01G016500 | 6276526-6282945(-) | Two-component response regulator |
| TraesNOR6D01G016600 | 6295388-6297707(+) | Cytochrome P450 709B2 |
| TraesNOR6D01G016700 | 6336395-6337459(-) | Extradiol ring-cleavage dioxygenase |
| TraesNOR6D01G016800 | 6340379-6342190(+) | Flavonoid 3'-monooxygenase CYP75B4 |
| TraesNOR6D01G016900 | 6340379-6342190(+) | Flavonoid 3'-monooxygenase CYP75B4 |
| TraesNOR6D01G017000 | 6357771-6359071(-) | Isoflavone reductase homolog IRL |
| TraesNOR6D01G017200 | 6394673-6395143(+) | Receptor-like protein EIX2 |
| TraesNOR6D01G017300 | 6472139-6473866(+) | Receptor-like protein EIX2 |
| TraesNOR6D01G017400 | 6472139-6491116(+) | Receptor-like protein EIX2 |
| TraesNOR6D01G017500 | 6473924-6475033(+) | Receptor-like protein EIX2 |
| TraesNOR6D01G017700 | 6490376-6491116(+) | Receptor-like protein EIX2 |
| TraesNOR6D01G017800 | 6509503-6512125(+) | Receptor-like protein EIX2 |
| Kariega: |  |  |
| TraesKAR6D01G0000860 | 1806956-1808724(+) | Isoflavone reductase homolog IRL |
| TraesKAR6D01G0000870 | 1823741-1825979(-) | Flavonoid 3'-monooxygenase CYP75B4 |
| TraesKAR6D01G0000880 | 1828638-1830374(+) | Extradiol ring-cleavage dioxygenase |
| TraesKAR6D01G0000960 | 1867275-1870490(-) | Cytochrome P450 709B2 |
| TraesKAR6D01G0000980 | 1893548-1907883(+) | Two-component response regulator |
| TraesKAR6D01G0001000 | 1968183-1969672(-) | Tyrosine/DOPA decarboxylase 3 |
| TraesKAR6D01G0001010 | 1986143-1989585(+) | O-acyltransferase WSD1 |
| TraesKAR6D01G0001030 | 2009674-2043830(+) | Two-component response regulator ORR21 |
| TraesKAR6D01G0001060 | 2083678-2084571(+) | Retrovirus-related Pol polyprotein from transposon RE1 |
| TraesKAR6D01G0001070 | 2138368-2145634(-) | MADS-box transcription factor 56 |
| TraesKAR6D01G0001080 | 2154977-2156001(+) | Ribosome-inactivating protein 9 |
| TraesKAR6D01G0001220 | 2269022-2272079(+) | Retrovirus-related Pol polyprotein from transposon TNT |
| TraesKAR6D01G0001260 | 2335549-2337457(+) | Tyrosine/DOPA decarboxylase 3 |
| TraesKAR6D01G0001340 | 2451811-2456333(+) | Retrovirus-related Pol polyprotein from transposon TNT |
| **TraesKAR6D01G0001420** | **2545418-2546220(+)** | **Disease resistance protein PIK6-NP** |
| TraesKAR6D01G0001550 | 2860518-2864097(+) | Retrovirus-related Pol polyprotein from transposon TNT |
| TraesKAR6D01G0001670 | 2979495-2983527(-) | Cysteine-rich receptor-like protein kinase 6 |
| TraesKAR6D01G0001690 | 3005860-3009735(-) | Copia protein [UniProtKB/Swiss-Prot:P04146] |
| TraesKAR6D01G0001730 | 3062975-3066951(+) | Cysteine-rich receptor-like protein kinase 6 |
| **TraesKAR6D01G0001810** | **3172892-3180080(+)** | **Disease resistance protein Piks-2** |
| TraesKAR6D01G0001820 | 3188336-3191998(+) | Retrovirus-related Pol polyprotein from transposon |
| TraesKAR6D01G0001840 | 3206377-3207147(+) | Retrovirus-related Pol polyprotein from transposon |
| TraesKAR6D01G0001880 | 3224937-3226171(-) | Retrovirus-related Pol polyprotein from transposon |
| **TraesKAR6D01G0001960** | **3340095-3344391(+)** | **Disease resistance protein RGA5** |
| **TraesKAR6D01G0001980** | **3354708-3388883(+)** | **Disease resistance protein RGA5** |
| **TraesKAR6D01G0002030** | **3452327-3458816(-)** | **Disease resistance protein RGA5** |
| **TraesKAR6D01G0002040** | **3470301-3478526(-)** | **Disease resistance protein RGA5** |
| Renan: |  |  |
| **TraesRN6D0100028500** | **4734719-4742799(+)** | **Disease resistance protein RGA5** |
| **TraesRN6D0100028600** | **4734736-4735733(+)** | **Disease resistance protein PIK6-NP** |
| **TraesRN6D0100028700** | **4754316-4760304(+)** | **Disease resistance protein RGA5** |
| TraesRN6D0100028800 | 4766551-4770275(-) | NA |
| **TraesRN6D0100028900** | **4811459-5398966(-)** | **Disease resistance protein RGA5** |
| **TraesRN6D0100029000** | **4811549-4943789(-)** | **Disease resistance protein RGA5** |
| **TraesRN6D0100029100** | **4812659-5398914(-)** | **Disease resistance protein Piks-2** |
| **TraesRN6D0100029500** | **4824659-4828378(-)** | **Disease resistance protein RGA5** |
| **TraesRN6D0100029700** | **4936744-4943778(-)** | **Disease resistance protein RGA5** |
| **TraesRN6D0100029800** | **4939057-4943655(-)** | **Disease resistance protein RGA5** |
| **TraesRN6D0100029900** | **4953497-4958281(-)** | **Disease resistance protein RGA5** |
| TraesRN6D0100030100 | 5033205-5033696(-) | NA |
| TraesRN6D0100030300 | 5326464-5334293(-) | NA |
| TraesRN6D0100030500 | 5408775-5409336(-) | NA |
| TraesRN6D0100030600 | 5439970-5443716(-) | Cysteine-rich receptor-like protein kinase 6 |
| TraesRN6D0100030700 | 5455614-5457953(+) | NA |
| TraesRN6D0100031000 | 5523145-5527446(+) | Cysteine-rich receptor-like protein kinase 6 |
| TraesRN6D0100031600 | 5934966-5937517(+) | NA |
| TraesRN6D0100032900 | 6048252-6048973(+) | NA |
| TraesRN6D0100033000 | 6054212-6055524(-) | NA |
| TraesRN6D0100033200 | 6118010-6120785(-) | NA |
| TraesRN6D0100033300 | 6148655-6150270(-) | Tyrosine/DOPA decarboxylase 3 |
| TraesRN6D0100033400 | 6303402-6306555(-) | MKI67 FHA domain-interacting nucleolar phosphoprotein |
| TraesRN6D0100033500 | 6307911-6309351(-) | NA |
| TraesRN6D0100033600 | 6314297-6314964(-) | NA |
| TraesRN6D0100033900 | 6328311-6330152(-) | Ribosome-inactivating protein 9 |
| TraesRN6D0100034000 | 6339609-6346720(+) | MADS-box transcription factor 56 |
| TraesRN6D0100034200 | 6432777-6459864(-) | Two-component response regulator ORR21 |
| TraesRN6D0100034300 | 6479874-6482895(-) | O-acyltransferase WSD1 |
| TraesRN6D0100034700 | 6577775-6584193(-) | Two-component response regulator |
| TraesRN6D0100034800 | 6596356-6599183(+) | Cytochrome P450 709B2 |
| TraesRN6D0100034900 | 6614130-6617227(+) | NA |
| TraesRN6D0100035200 | 6636683-6638659(-) | Extradiol ring-cleavage dioxygenase |
| TraesRN6D0100035300 | 6641264-6643313(+) | Flavonoid 3'-monooxygenase CYP75B4 |
| TraesRN6D0100035400 | 6650210-6650865(+) | Flavonoid 3'-monooxygenase CYP75B4 |
| TraesRN6D0100035500 | 6664520-6666221(-) | Isoflavone reductase homolog IRL |
| TraesRN6D0100035600 | 6672999-6675287(-) | NA |
| TraesRN6D0100035700 | 6675517-6676475(+) | Receptor-like protein EIX2 |
| TraesRN6D0100035900 | 6691749-6694648(+) | Receptor-like protein EIX2 |
| TraesRN6D0100036700 | 6746088-6817827(+) | Receptor-like protein EIX2 |
| TraesRN6D0100036600 | 6746088-6747814(+) | Receptor-like protein EIX2 |
| TraesRN6D0100036800 | 6747873-6748981(+) | Receptor-like protein EIX2 |
| TraesRN6D0100037000 | 6761955-6785654(+) | LRR receptor-like serine/threonine-protein kinase FLS2 |
| TraesRN6D0100036900 | 6761955-6763816(+) | Probable inactive leucine-rich repeat receptor kinase XIAO |
| SY Mattis: |  |  |
| TraesSYM6D01G000100 | 13563-15041(+) | Tyrosine/DOPA decarboxylase 3 |
| TraesSYM6D01G000200 | 42395-45173(+) | NA |
| TraesSYM6D01G000300 | 107360-108646(+) | NA |
| TraesSYM6D01G000400 | 113882-114604(-) | NA |
| TraesSYM6D01G000500 | 226086-228297(-) | NA |
| TraesSYM6D01G000600 | 603954-607986(-) | Cysteine-rich receptor-like protein kinase 6 |
| TraesSYM6D01G000700 | 673407-674818(-) | NA |
| TraesSYM6D01G000800 | 687451-690862(+) | Cysteine-rich receptor-like protein kinase 6 |
| TraesSYM6D01G000900 | 720638-721198(+) | NA |
| TraesSYM6D01G001100 | 885261-885753(+) | NA |
| **TraesSYM6D01G001200** | **985146-988848(+)** | **Disease resistance protein RGA5** |
| TraesSYM6D01G001300 | 1043286-1047058(+) | NA |
| **TraesSYM6D01G001400** | **1055143-1058841(-)** | **Disease resistance protein RGA5** |
| **TraesSYM6D01G001500** | **1071606-1080222(-)** | **Disease resistance protein RGA5** |
| TraesSYM6D01G001600 | 1198279-1200583(-) | Probable nucleolar protein 5-1 |
| TraesSYM6D01G001700 | 1296135-1297468(-) | WAT1-related protein At3g30340 |
| TraesSYM6D01G001800 | 1356595-1357404(+) | NA |
| TraesSYM6D01G001900 | 1360178-1361971(+) | Ferredoxin--NADP reductase, leaf isozyme 1, chloroplastic |
| TraesSYM6D01G002000 | 1412531-1414344(+) | Bidirectional sugar transporter SWEET14 |
| TraesSYM6D01G002100 | 1452363-1454258(+) | Bidirectional sugar transporter SWEET14 |
| TraesSYM6D01G002200 | 1465132-1466816(+) | Bidirectional sugar transporter SWEET14 |
| TraesSYM6D01G002300 | 1477498-1479168(+) | Bidirectional sugar transporter SWEET14 |
| TraesSYM6D01G002500 | 1552887-1554421(-) | WAT1-related protein At3g30340 |
| TraesSYM6D01G002600 | 1555336-1557164(-) | WAT1-related protein At3g30340 |
| TraesSYM6D01G002700 | 1573345-1574610(-) | NA |
| TraesSYM6D01G002800 | 1588373-1592190(+) | Lipoxygenase 2.2, chloroplastic |
| TraesSYM6D01G002900 | 1624762-1648491(+) | NA |
| TraesSYM6D01G003000 | 1661561-1662519(+) | NA |
| TraesSYM6D01G003100 | 1667976-1668467(+) | NA |
| TraesSYM6D01G003200 | 1672207-1672608(+) | NA |
| TraesSYM6D01G003300 | 1709287-1713549(-) | NA |
| TraesSYM6D01G003500 | 1725544-1726041(-) | Putative Polyprotein CP |
| TraesSYM6D01G003600 | 1765185-1793798(-) | F-box protein At5g03970 |
| TraesSYM6D01G003700 | 1861340-1862387(+) | Protein LIAT1 |
| TraesSYM6D01G003800 | 1866735-1869595(+) | NA |
| TraesSYM6D01G003900 | 1897063-1899674(-) | NA |
| TraesSYM6D01G004000 | 1912088-1915340(-) | NA |
| TraesSYM6D01G004100 | 1922528-1923130(+) | Putative invertase inhibitor |
| TraesSYM6D01G004200 | 1923856-1924467(+) | Putative invertase inhibitor |
| TraesSYM6D01G004300 | 1924844-1927343(-) | DNA-directed RNA polymerase III subunit 2 |
| **TraesSYM6D01G004500** | **2036029-2042850(+)** | **Disease resistance protein Piks-2** |
| TraesSYM6D01G004600 | 2148069-2151591(+) | Probable transcription factor KAN2 |
| TraesSYM6D01G004700 | 2259094-2264883(+) | Extra-large guanine nucleotide-binding protein 3 |
| TraesSYM6D01G004800 | 2276312-2277841(+) | NA |
| TraesSYM6D01G004900 | 2306747-2307136(-) | NA |
| TraesSYM6D01G005000 | 2306756-2307136(-) | NA |
| TraesSYM6D01G005100 | 2327212-2329476(+) | NA |
| TraesSYM6D01G005200 | 2444418-2444813(+) | NA |
| TraesSYM6D01G005300 | 2449051-2451594(+) | Probable E3 ubiquitin-protein ligase WAVH2 |
| TraesSYM6D01G005400 | 2455163-2456506(-) | NA |
| TraesSYM6D01G005500 | 2461262-2465382(-) | RuBisCO large subunit-binding protein subunit beta, chloroplastic |
| TraesSYM6D01G005600 | 2561281-2564321(+) | Red chlorophyll catabolite reductase 1, chloroplastic |
| TraesSYM6D01G005700 | 2569668-2571283(+) | GDSL esterase/lipase At1g28580 |
| TraesSYM6D01G005800 | 2576041-2576655(+) | Red chlorophyll catabolite reductase (Fragment) |
| TraesSYM6D01G005900 | 2581705-2582067(+) | NA |
| TraesSYM6D01G006000 | 2589978-2590473(+) | Thiosulfate sulfurtransferase 18 |
| TraesSYM6D01G006100 | 2592850-2593344(+) | Rhodanese-like domain-containing protein 17 |
| TraesSYM6D01G006200 | 2607320-2607821(+) | Rhodanese-like domain-containing protein 17 |
| TraesSYM6D01G006300 | 2631843-2632336(+) | Rhodanese-like domain-containing protein 17 |
| TraesSYM6D01G006400 | 2706387-2708465(+) | Tricetin 3',4',5'-O-trimethyltransferase |
| TraesSYM6D01G006600 | 2735410-2738699(-) | Flavonoid 3',5'-hydroxylase 2 |
| TraesSYM6D01G006700 | 2776212-2780641(-) | T-complex protein 1 subunit theta |
| TraesSYM6D01G006800 | 2834163-2834544(-) | NA |
| TraesSYM6D01G006900 | 2888695-2891558(-) | NA |
| TraesSYM6D01G007000 | 2907910-2909996(+) | Cysteine-rich receptor-like protein kinase 15 |
| TraesSYM6D01G007100 | 2973980-2976520(+) | NA |
| **TraesSYM6D01G007200** | **2997675-3003192(+)** | **Protein ENHANCED DISEASE RESISTANCE 2** |
| TraesSYM6D01G007300 | 3010489-3012993(+) | Crocetin glucosyltransferase, chloroplastic |
| TraesSYM6D01G007500 | 3024289-3025394(+) | Pathogen-related protein |
| TraesSYM6D01G007600 | 3032644-3035412(+) | Immune-associated nucleotide-binding protein 9 |
| TraesSYM6D01G007700 | 3055637-3068872(+) | Immune-associated nucleotide-binding protein 9 |
| TraesSYM6D01G007800 | 3090522-3093045(+) | Multiple organellar RNA editing factor 2, chloroplastic |
| TraesSYM6D01G007900 | 3126549-3128185(-) | Patatin-like protein 2 |
| TraesSYM6D01G008000 | 3133854-3136578(-) | NA |
| TraesSYM6D01G008100 | 3149190-3151636(-) | NA |
| TraesSYM6D01G008200 | 3157002-3160051(-) | Phytochrome-interacting ankyrin-repeat protein 1 |
| TraesSYM6D01G008300 | 3169955-3171280(-) | F-box protein At5g07610 |
| TraesSYM6D01G008400 | 3175352-3192154(+) | Achilleol B synthase |
| TraesSYM6D01G008500 | 3213909-3215103(-) | NA |
| TraesSYM6D01G008600 | 3227085-3229139(-) | NA |
| TraesSYM6D01G008700 | 3375283-3376527(+) | NA |
| TraesSYM6D01G008800 | 3431198-3432496(-) | NA |
| TraesSYM6D01G008900 | 3525447-3526637(-) | Receptor-like protein EIX2 |
| TraesSYM6D01G009000 | 3526700-3528265(-) | Receptor-like protein EIX2 |
| TraesSYM6D01G009100 | 3539519-3542326(+) | Receptor-like protein EIX2 |
| TraesSYM6D01G009200 | 3604733-3605226(-) | NA |
| TraesSYM6D01G009300 | 3617653-3617997(-) | NA |
| TraesSYM6D01G009400 | 3634906-3644137(-) | E3 ubiquitin-protein ligase UPL4 |
| TraesSYM6D01G009500 | 3686698-3688641(+) | NA |
| TraesSYM6D01G009600 | 3686698-3688641(+) | NA |
| TraesSYM6D01G009700 | 3706262-3708202(+) | NA |
| **TraesSYM6D01G009800** | **3717349-3721351(+)** | **Disease resistance protein RGA5** |
| TraesSYM6D01G009900 | 3762731-3767810(+) | Protein disulfide isomerase-like 1-4 |
| TraesSYM6D01G010000 | 3762740-3767810(+) | Protein disulfide isomerase-like 1-4 |
| TraesSYM6D01G010100 | 3768082-3769558(-) | NA |
| TraesSYM6D01G010200 | 3770557-3772351(-) | Ferredoxin-thioredoxin reductase catalytic chain, chloroplastic |
| TraesSYM6D01G010300 | 3773282-3776981(-) | NA |
| TraesSYM6D01G010400 | 3791915-3795773(+) | FAM10 family protein At4g22670 |
| TraesSYM6D01G010500 | 3797848-3838111(-) | Protein disulfide isomerase-like 1-4 |
| TraesSYM6D01G010600 | 3867367-3869291(-) | Protein disulfide isomerase-like 1-4 |
| TraesSYM6D01G010800 | 3930175-3931491(+) | Leucoanthocyanidin dioxygenase 1 |
| TraesSYM6D01G010900 | 3935689-3941232(-) | NA |
| TraesSYM6D01G011000 | 3961989-3968642(+) | Probable LRR receptor-like serine/threonine-protein kinase At1g51810 |
| TraesSYM6D01G011100 | 4012915-4019828(-) | Probable LRR receptor-like serine/threonine-protein kinase At1g51810 |
| TraesSYM6D01G011200 | 4040342-4041292(-) | NA |
| TraesSYM6D01G011300 | 4048982-4049492(+) | NA |
| TraesSYM6D01G011400 | 4087025-4087954(+) | NA |
| TraesSYM6D01G011500 | 4140282-4141205(+) | NA |
| TraesSYM6D01G011600 | 4173857-4186273(+) | NA |
| TraesSYM6D01G011700 | 4194746-4195680(+) | NA |
| TraesSYM6D01G011800 | 4210872-4214527(-) | Translation initiation factor IF3-1, mitochondrial |
| TraesSYM6D01G011900 | 4219051-4223696(+) | Amino acid transporter AVT1C |
| TraesSYM6D01G012000 | 4232123-4233055(-) | NA |
| TraesSYM6D01G012100 | 4342791-4343702(-) | NA |
| TraesSYM6D01G012200 | 4350886-4351841(-) | NA |
| TraesSYM6D01G012300 | 4401705-4402934(-) | GDSL esterase/lipase EXL3 |
| TraesSYM6D01G012400 | 4418091-4418519(+) | NA |
| TraesSYM6D01G012500 | 4450064-4451209(+) | SKP1-like protein 4 |
| TraesSYM6D01G012600 | 4471822-4481518(+) | Wall-associated receptor kinase 5 |
| TraesSYM6D01G012700 | 4471822-4481518(+) | Wall-associated receptor kinase 5 |
| TraesSYM6D01G013100 | 4588573-4590112(+) | Receptor kinase-like protein Xa21 |
| TraesSYM6D01G013200 | 4628086-4629252(-) | NA |
| TraesSYM6D01G013300 | 4790628-4791281(-) | Wall-associated receptor kinase 4 |
| TraesSYM6D01G013400 | 4799614-4800069(+) | Alpha-amylase inhibitor 0.28 |
| TraesSYM6D01G013500 | 4900229-4901194(-) | Wall-associated receptor kinase 1 |
| TraesSYM6D01G013600 | 4966015-4966434(-) | NA |
| TraesSYM6D01G013700 | 5088169-5089425(+) | SKP1-like protein 1B |
| TraesSYM6D01G013800 | 5090103-5092498(+) | Glycerate dehydrogenase HPR, peroxisomal |
| TraesSYM6D01G013900 | 5249754-5250017(+) | NA |
| TraesSYM6D01G014000 | 5253169-5253690(-) | NA |
| TraesSYM6D01G014100 | 5352353-5352901(+) | NA |
| TraesSYM6D01G014200 | 5439619-5440173(+) | NA |
| TraesSYM6D01G014300 | 5464132-5465460(+) | Protein Rf1, mitochondrial |
| TraesSYM6D01G014400 | 5465513-5466277(+) | Protein Rf1, mitochondrial |
| **TraesSYM6D01G014500** | **5538171-5541833(+)** | **Disease resistance protein Piks-1** |
| **TraesSYM6D01G014600** | **5538171-5541833(+)** | **Disease resistance protein Piks-1** |
| **TraesSYM6D01G014700** | **5538535-5548164(-)** | **Disease resistance protein Piks-2** |
| TraesSYM6D01G014800 | 5562129-5563574(-) | Protein Rf1, mitochondrial |
| TraesSYM6D01G014900 | 5568403-5570103(-) | Protein Rf1, mitochondrial |
| TraesSYM6D01G015000 | 5574235-5574816(+) | NA |
| TraesSYM6D01G015100 | 5609814-5610433(-) | NA |
| **TraesSYM6D01G015200** | **5692944-5695328(+)** | **Disease resistance protein RGA5** |
| **TraesSYM6D01G015300** | **5700838-5704872(+)** | **Disease resistance protein RGA5** |
| TraesSYM6D01G015400 | 5773167-5775428(+) | BURP domain-containing protein 14 |
| TraesSYM6D01G015500 | 5820765-5821217(-) | NA |
| TraesSYM6D01G015600 | 5827008-5827418(+) | NA |
| TraesSYM6D01G015700 | 5875181-5875564(+) | Outer envelope protein 61 |
| TraesSYM6D01G015800 | 5875193-5875564(+) | NA |
| TraesSYM6D01G015900 | 5876769-5884404(-) | Ubiquitin-like domain-containing protein CIP73 |
| TraesSYM6D01G016000 | 5876781-5883383(-) | Ubiquitin-like domain-containing protein CIP73 |
| TraesSYM6D01G016100 | 5886555-5889050(-) | Putative nitric oxide synthase |
| TraesSYM6D01G016200 | 5894959-5898454(-) | Wall-associated receptor kinase 5 |
| TraesSYM6D01G016300 | 5951692-5954382(-) | Receptor-like protein EIX2 |
| TraesSYM6D01G016400 | 6003173-6004478(-) | Probable glutathione S-transferase GSTF1 |
| TraesSYM6D01G016500 | 6006575-6009304(+) | Receptor-like protein 43 |
| TraesSYM6D01G016600 | 6016055-6022401(+) | NA |
| TraesSYM6D01G016700 | 6154849-6157036(-) | NA |
| TraesSYM6D01G016800 | 6398350-6401197(+) | Trihelix transcription factor DF1 |
| TraesSYM6D01G016900 | 6473024-6475648(-) | Receptor-like protein EIX2 |
| TraesSYM6D01G017000 | 6528589-6530451(-) | Probable inactive leucine-rich repeat receptor kinase XIAO |
| TraesSYM6D01G017100 | 6543513-6544622(-) | Receptor-like protein EIX2 |
| TraesSYM6D01G017200 | 6544680-6546407(-) | Receptor-like protein EIX2 |
| TraesSYM6D01G017300 | 6598019-6600919(-) | Receptor-like protein EIX2 |
| TraesSYM6D01G017400 | 6616420-6617160(-) | Receptor-like protein EIX2 |
| TraesSYM6D01G017500 | 6617608-6619629(-) | LRR receptor-like serine/threonine-protein kinase FLS2 |
| TraesSYM6D01G017600 | 6626715-6628016(+) | Isoflavone reductase homolog IRL |
| TraesSYM6D01G017700 | 6641928-6642584(-) | Flavonoid 3'-monooxygenase CYP75B4 |
| TraesSYM6D01G017800 | 6649703-6651509(-) | Flavonoid 3'-monooxygenase CYP75B4 |
| TraesSYM6D01G017900 | 6654193-6655417(+) | Extradiol ring-cleavage dioxygenase |
| TraesSYM6D01G018000 | 6694032-6696351(-) | Cytochrome P450 709B2 |
| TraesSYM6D01G018100 | 6708645-6715064(+) | Two-component response regulator ORR24 |
| TraesSYM6D01G018200 | 6720690-6721328(+) | NA |
| TraesSYM6D01G018300 | 6809107-6811997(+) | O-acyltransferase WSD1 |
| TraesSYM6D01G018400 | 6832505-6858376(+) | Two-component response regulator ORR21 |
| TraesSYM6D01G018500 | 6945303-6952048(-) | MADS-box transcription factor 56 |
| TraesSYM6D01G018600 | 6961794-6962570(+) | Ribosome-inactivating protein 9 |
| TraesSYM6D01G018700 | 6977037-6977705(+) | NA |
| TraesSYM6D01G018800 | 6983222-6983767(+) | NA |
| TraesSYM6D01G018900 | 6986344-6988376(+) | MKI67 FHA domain-interacting nucleolar phosphoprotein |
|  |  |  |
